# A BOHB-optimized stacking ensemble approach for predicting the coefficient of compressibility and quantifying uncertainty of guilin lateritic clays

**DOI:** 10.1016/j.isci.2026.116946

**Published:** 2026-07-29

**Authors:** Tongtong Wang, Hanying Bai, Jinghe Li

**Affiliations:** 1 Guangxi Key Laboratory of Hidden Metallic Ore Deposits Exploration, Guilin University of Technology, Guilin 541006, China; 2 School of Earth Sciences, Guilin University of Technology, Guilin 541006, China

**Keywords:** guilin lateritic clay, coefficient of compressibility, stacking ensemble, interpretability analysis, uncertainty quantification

## Abstract

The coefficient of compressibility of lateritic clays is central to settlement analysis and foundation design, yet rapid assessment is hampered by their paradoxical combination of high plasticity and low-to-medium compressibility. A Bayesian Optimization with HyperBand (BOHB)-optimized stacking ensemble (CatBoost-XGBoost-LightGBM + ElasticNet) predicts this coefficient from basic physical indices using 454 datasets from Guilin, China. The model achieves a test R^2^ of 0.9692 and RMSE of 0.0183 MPa^−1^, outperforming all individual base models. SHAP and partial dependence analyses reveal that liquidity index and water content dominate the prediction, accounting for over 95% of feature importance, with their synergistic elevation as the primary driver of high compressibility. Spatial cross-validation yields a mean test R^2^ of 0.9415, and Monte Carlo simulation confirms robustness: 80% of samples yield a 90% confidence interval width below 0.05 MPa^−1^. This framework enables rapid, reliable compressibility assessment for lateritic clays.

## Highlights

• A BOHB-stacking ensemble predicts compressibility of regional lateritic clays
• SHAP analyses reveal synergistic effects of liquidity index and water content
• Spatial cross-validation shows performance bounds on unseen sites
• A workflow integrating ensemble prediction, interpretation, and uncertainty

## Graphical abstract

**Figure:**
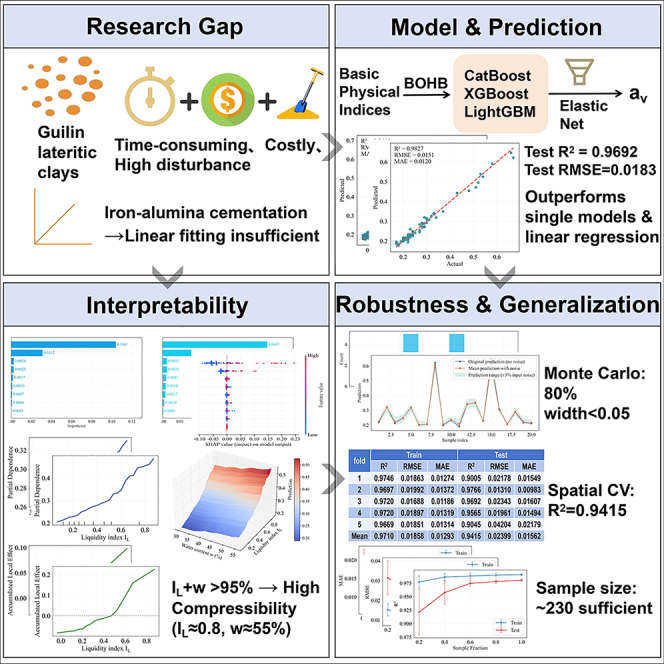

## Teaser

Geology; Computer science; Engineering

## Introduction

The coefficient of compressibility (a_v_) is central to settlement calculation and foundation design; its accuracy directly affects the long-term service safety of major infrastructure.^1^ The global boom in large-scale infrastructure construction demands ever more accurate characterization of foundation soil behavior.^2^ Yet conventional oedometer tests take 7–10 days, are expensive, and disturb samples—especially for structured clays—so laboratory parameters systematically deviate from *in situ* results.^3^^,^^4^ More fundamentally, soils are inherently highly variable in space, and a few boreholes rarely capture the full spatial distribution of compressibility across a site.^5^^,^^6^ This problem is particularly acute for lateritic clays. These soils have high liquid limits, high plasticity, and high void ratios, yet typically exhibit low to medium compressibility, a combination that complicates the inference of compressibility from basic physical indices.

Lateritic clays are widespread across southern China, Southeast Asia, South America, and Africa, among other tropical and subtropical regions. Their main engineering features include high liquid limits, high plasticity, high void ratios coexisting with low-to-medium compressibility, and pronounced shrinkage cracking.^7^^,^^8^ In South China, lateritic clays cover more than 300,000 km^2^ and form the primary foundation stratum for regional transport and building infrastructure.^9^^,^^10^ Guilin, located in a typical karst area within this region, has seen intensive construction of high-rise buildings, expressways, and high-speed railways, which impose stringent demands on settlement assessment accuracy for lateritic clay foundations.^11^ During laterization, Guilin lateritic clays developed a stable aggregate structure cemented by free iron oxides.^12^^,^^13^ Consequently, their mechanical behavior differs markedly from that of ordinary cohesive soils: High plasticity does not necessarily imply high compressibility; instead, the cementation actually strengthens the soil skeleton’s resistance to deformation.^14^^,^^15^ As a result, the relationship between basic physical indices and compressibility in these soils is more complex than in ordinary clays. Meanwhile, the thickness of Guilin lateritic clay layers is strongly controlled by the undulating karst bedrock surface, causing severe spatial variability. Limited boreholes cannot fully reveal the site-specific distribution of compressibility; laboratory oedometer tests are slow and costly, and *in situ* methods lack empirical conversion bases tailored to lateritic clays. Thus, rapidly deriving the coefficient of compressibility from easily measured physical indices naturally becomes an option that balances efficiency and economy. However, the nonlinear relationships introduced by the iron-aluminum cementation structure lead to large deviations under conventional regression methods, calling for data-driven approaches capable of capturing this complex mapping.

In recent years, data-driven methods have opened new avenues for tackling the challenges of geotechnical parameter prediction. Within the broader geotechnical domain, boosting algorithms—XGBoost, CatBoost, and LightGBM, among others—have been increasingly applied across diverse tasks, from predicting soil activity and slope stability to assessing rockburst susceptibility.^16^^,^^17^^,^^18^ Meanwhile, ensemble techniques such as super-learner, stacking, and stacked generalization have been adopted to further enhance predictive accuracy and robustness by fusing complementary models.^19^^,^^20^ A focused body of research has specifically targeted soil compressibility prediction—hybrid genetic programming with XGBoost for the compression index of clayey soils, and boosting-based interpretable frameworks for Gulf Coast clays.^21^^,^^22^ These studies consistently demonstrate that boosting-based models outperform conventional statistical regression in capturing the nonlinear mapping between basic physical indices and compressibility. At the same time, the research paradigm is shifting from a narrow focus on accuracy toward interpretability and uncertainty quantification. SHAP analysis is now widely used to identify key controlling factors,^23^^,^^24^ while Monte Carlo simulation and Bayesian inference serve to quantify predictive uncertainty.^25^^,^^26^ Together, these advances outline a full intelligent prediction framework that integrates hybrid optimization, interpretable analysis, and uncertainty quantification.^27^^,^^28^^,^^29^ Nevertheless, applying this framework directly to predict the coefficient of compressibility of Guilin lateritic clays faces three levels of challenges: prediction target, regional adaptation, and method integration. On the prediction target level, most existing studies focus on the compression index (Cc), whereas engineering settlement calculations directly use the coefficient of compressibility (a_v_).^30^^,^^31^ The compression curve of lateritic clays remains relatively steep even at higher pressures, so the empirical conversion between Cc and a_v_ may be invalid. On the regional adaptation level, existing models are largely built on general clays or global datasets, ignoring the unique iron-aluminum cementation microstructure of Guilin lateritic clays.^32^ This cementation means that high liquid limits and high plasticity do not necessarily translate into high compressibility; hence, the compressibility of highly plastic samples tends to be overestimated. On the method integration level, hyperparameter optimization, multi-model ensembling, interpretable analysis, and uncertainty quantification have each become relatively mature, yet examples that integrate them into a complete evaluation workflow tailored to a regionally distinctive soil remain scarce.^33^

To systematically address the above challenges—namely, the lack of predictive capability for the coefficient of compressibility a_v_ in existing models, insufficient characterization of regional lateritic clay features, and the absence of systematic integration along the methodological chain—this study constructs a three-layer progressive analytical framework comprising ensemble prediction, interpretable analysis, and uncertainty quantification. Driven by 454 sets of geotechnical test data on lateritic clays from the Guilin area, the framework aims to answer the following questions: Can a high-precision prediction model for a_v_ be established based on basic physical indices? What are the dominant physical factors controlling the compressibility of lateritic clays, and what is their synergistic mechanism? Does the model’s robustness and generalization capability under measurement errors and unsurveyed site conditions meet engineering application requirements?

Concretely, this study first adopts a stacking framework to integrate multiple gradient boosting tree models. After joint optimization via Bayesian Optimization with HyperBand (BOHB), the framework establishes a nonlinear mapping from physical indices to the coefficient of compressibility a_v_. Stacking fuses the complementary strengths of heterogeneous models through a meta-learner, compensating for the predictive deficiencies of any single model. Second, SHAP values and accumulated local effects (ALEs) are introduced to quantify the marginal contribution of each feature, and three-dimensional partial dependence plots are used to identify the dominant controlling factors and critical thresholds at which the compressibility abruptly changes. SHAP values overcome the limitation of conventional methods in capturing nonlinear effects, while ALEs avoid the extrapolation bias caused by feature correlation in partial dependence plots. Together, they enhance the characterization of regional lateritic clay features. Finally, Monte Carlo simulation is employed to test the model’s robustness against measurement errors, and spatial cross-validation combined with sample size sensitivity analysis is used to evaluate its generalization capability. Spatial cross-validation partitions the data by site groups, correcting the tendency of random cross-validation to overestimate performance by ignoring spatial correlations, thereby closing the methodological loop. The significance of this study lies not only in providing an accuracy-controllable and fully interpretable rapid assessment method for the coefficient of compressibility of Guilin lateritic clays, but also in establishing a reproducible full-chain technical paradigm of “ensemble prediction—interpretable analysis—uncertainty quantification,” offering a methodological framework for the intelligent assessment of regional special soil parameters under analogous geological conditions.

## Results

### Exploratory data analysis

**Figure 1:**
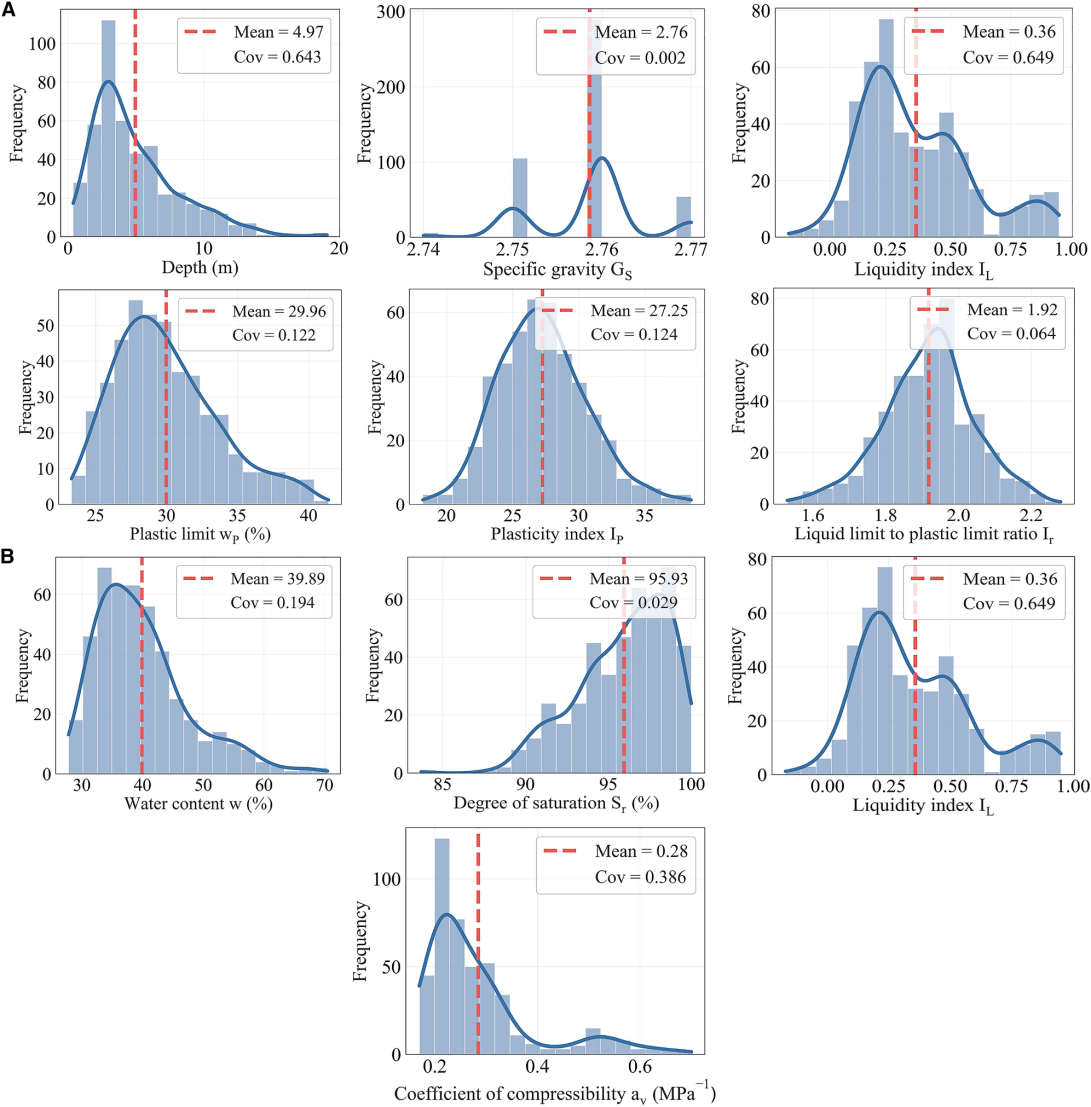
Statistical histograms of input and output variables (A) Physical indices: depth (D), specific gravity (Gs), liquid limit (w_L_), plastic limit (w_P_), plasticity index (I_P_), and liquid limit to plastic limit ratio (Ir). Each panel presents a histogram with an overlaid kernel density curve. (B) State and compressibility indices: water content (w), degree of saturation (Sr), liquidity index (I_L_), and coefficient of compressibility (a_v_). Each panel presents a histogram with an overlaid kernel density curve. Data are based on 454 samples (*n* = 454). See also Table 1.

**Figure 2:**
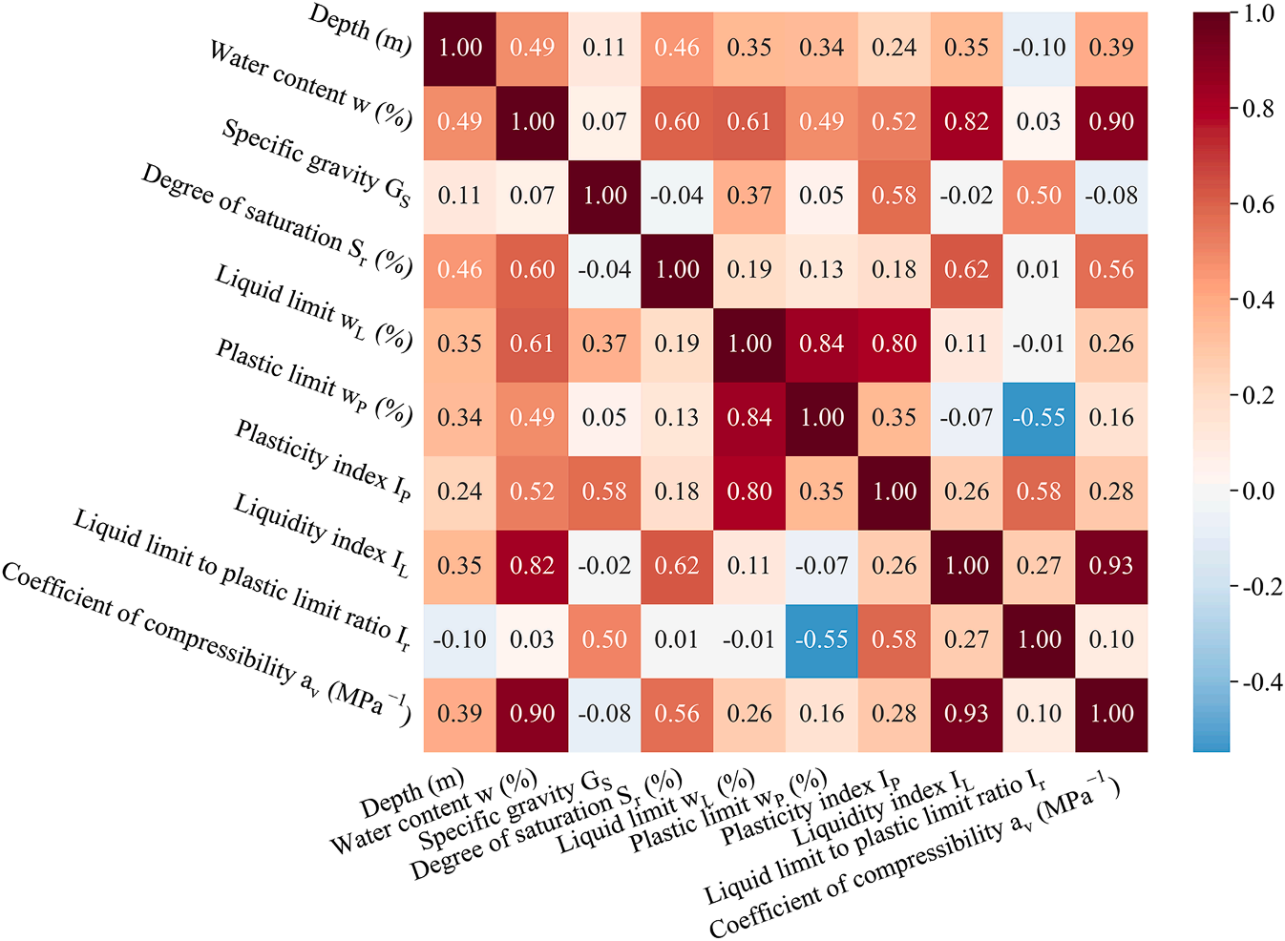
Correlation heatmap of all variables Pearson’s correlation coefficients are shown, ranging from −1 to +1. Data are based on 454 samples (*n* = 454).

The basic statistical characteristics of the dataset are presented in Table 1, Figures 1, and 2. Table 1 summarizes the statistical characteristics of each variable, including minimum, maximum, mean, standard deviation, coefficient of variation (Cov), skewness, and interquartile range (IQR). Figure 1 presents the distribution shape of each variable through histograms and probability density curves, while Figure 2 reveals the linear correlations among variables via a correlation heatmap.

**Table 1:** Statistical description of input and output variables

Variables	Unit	Min	Max	Mean	Std	Cov	Skewness	IQR
D	m	0.4	19.1	4.97	3.20	0.643	1.28	3.875
w	%	27.8	70.5	39.89	7.73	0.194	1.14	9.325
Gs	–	2.74	2.77	2.76	0.006	0.002	−0.23	0
Sr	%	83.7	100	95.93	2.74	0.029	−0.78	4
w_L_	%	45.3	78.3	57.21	5.76	0.100	0.69	7.875
w_P_	%	23.3	41.4	29.96	3.65	0.122	0.71	4.575
I_P_	–	18.2	38.5	27.25	3.37	0.124	0.39	4.40
I_L_	–	−0.17	0.95	0.36	0.23	0.649	0.78	0.3
Ir	–	1.53	2.28	1.92	0.12	0.064	−0.17	0.140
a_v_	MPa^−1^	0.17	0.7	0.28	0.11	0.386	1.74	0.1

Data are based on 454 samples (*n* = 454). Cov, coefficient of variation; IQR, interquartile range. See also Figure 1.

From the mean values in Table 1, the Guilin lateritic clays represented by the study dataset exhibit typical high liquid limit and high plasticity characteristics: the mean liquid limit (w_L_) is 57.21%, and the mean plasticity index (I_P_) reaches 27.25%, both significantly higher than those of ordinary cohesive soils. The mean natural water content (w) is 39.89%, close to the liquid limit, indicating that the soils are mostly in a plastic to soft plastic state. The mean coefficient of compressibility (a_v_) is 0.28 MPa^−1^, falling into the medium compressibility category, but the maximum value reaches 0.70 MPa^−1^, suggesting that some samples are highly compressible. The coefficients of variation (Cov) of the variables show clear differences: sampling depth (D, Cov = 0.643) and liquidity index (I_L_, Cov = 0.649) exhibit high dispersion, indicating pronounced variations in terrain and moisture conditions across sites; in contrast, specific gravity (Gs, Cov = 0.002) and saturation (Sr, Cov = 0.029) show extremely low variability, reflecting relatively stable mineral composition and saturation status in lateritic clays. This disparity in dispersion offers important insights for subsequent modeling: for highly variable features, the model needs to capture local outliers; for low-variability features, the representation can be appropriately simplified to avoid overfitting.

Skewness coefficients reveal the asymmetry of each variable’s distribution. The coefficient of compressibility is strongly right-skewed (skewness = 1.74), indicating that most samples cluster in the lower compressibility range, with a few highly compressible samples that are particularly critical for engineering settlement control. Sampling depth is also right-skewed (skewness = 1.28), reflecting that shallow samples dominate while deep samples are relatively sparse. The liquidity index is right-skewed (skewness = 0.78), meaning most samples are in a plastic-to-stiff state, with only a few in a fluid plastic state. In contrast, saturation and the liquid-plastic ratio (Ir, defined as w_L_/w_P_) exhibit left-skewed distributions, suggesting that the soils are mostly near saturation and the liquid-plastic ratio is relatively concentrated. The IQR further quantifies the dispersion of the middle 50% of samples: The IQR of the liquidity index is only 0.3, while that of sampling depth reaches 3.875 m. This contrast confirms the dominant role of depth variation in the spatial variability of soil properties.

Figure 1 presents histograms and probability density curves, visually confirming the distribution shapes of each variable. Natural water content, liquid limit, plastic limit (w_P_), and plasticity index all exhibit approximately unimodal symmetric distributions, indicating that the physical properties of lateritic clays in the study area are statistically stable. In contrast, sampling depth displays a multimodal character, reflecting variations in stratum thickness across different engineering sites. The distribution of the coefficient of compressibility shows a pronounced long right tail, consistent with the skewness analysis. Notably, the liquidity index concentrates in the range of 0.2–0.5, while it appears sparsely in the negative region (stiff state) and above 0.75 (soft plastic to fluid plastic state). This pattern aligns with the engineering observation that Guilin lateritic clays are mostly in a plastic state.

Figure 2 is a correlation heatmap that reveals the underlying relationships among variables. A cluster of strong positive correlations exists among physical property indices: the correlation coefficients between liquid limit and plastic limit (w_L_-w_P_), between plasticity index and liquid limit (I_P_-w_L_), and between plasticity index and liquid-plastic ratio (I_P_-Ir) all exceed 0.85, reflecting the inherent consistency of plasticity characteristics in lateritic clays. Natural water content and liquidity index (w-I_L_) exhibit a strong positive correlation (>0.8), consistent with the fundamental soil mechanics principle that higher water content yields softer soil. The coefficient of compressibility (a_v_) correlates positively with natural water content and liquidity index, with coefficients exceeding 0.9, indicating that increased water content and softer soil dominate the increase in compressibility. Notably, the coefficient of compressibility shows only a weak positive correlation with plasticity index (0.28) and an extremely weak correlation with the liquid-plastic ratio (0.10). The liquid-plastic ratio reflects the width of the plastic range; its weak correlation with a_v_ indicates that the iron-aluminum cementation suppresses the compressibility otherwise associated with a wide plastic range. This suggests that, due to the unique iron-aluminum cementation structure and stable aggregate effects, the high liquid limit and high plasticity of lateritic clays do not directly translate into high compressibility.

In terms of negative correlations, plastic limit and liquid-plastic ratio (w_P_-Ir) exhibit a significant negative correlation (−0.55), indicating that a larger liquid-plastic ratio brings the soil closer to the plastic limit state. The coefficient of compressibility shows a weak negative correlation with specific gravity, but a moderate positive correlation with saturation (0.56), suggesting that higher saturation contributes modestly to compressibility, while the influence of mineral composition remains limited. The clustering structure among variables in the heatmap (a cluster of physical property indices and a cluster of state and compressibility indices) provides clear guidance for feature selection and multicollinearity diagnosis in subsequent modeling.

In summary, the data characteristics reveal several key issues in predicting the coefficient of compressibility of Guilin lateritic clays: The high variability of sampling depth and liquidity index demands that the model possess robust spatial extrapolation capability; the right-skewed distribution of the coefficient of compressibility requires the model to focus on prediction accuracy for highly compressible samples; the strong correlations among physical indices reflect the inherent interdependency of these basic soil properties and require consideration in both model construction and the interpretation of feature importance. The correlations between the coefficient of compressibility and key physical indices—consistent with soil mechanics principles—lay a foundation for constructing physically interpretable machine learning models. The above analysis provides critical prior information for subsequent model selection, hyperparameter optimization, and interpretability analysis. Based on these data characteristics, all input variables were standardized to zero mean and unit variance before modeling to eliminate scale differences among features.

### Evaluation of base models

To establish a nonlinear mapping between physical indices and the coefficient of compressibility a_v_, the predictive performance of individual models on a_v_ is first evaluated. XGBoost, CatBoost, LightGBM, random forest (RF), and deep neural networks (DNNs) are assessed through 30 independent runs. Key performance metrics include R^2^, RMSE, and MAE. Table 2 summarizes the mean values of these metrics on the training and test sets, ranked by test R^2^.

**Table 2:** Evaluation of base models

Base model	R^2^ (mean)		RMSE (mean)^a^		MAE (mean)^a^		Ranking (R^2^)
	Train	Test	Train	Test	Train	Test	
CatBoost	0.9884	0.9665	0.0116	0.0190	0.0083	0.0131	1
XGBoost	0.9883	0.9600	0.0117	0.0209	0.0079	0.0142	2
LightGBM	0.9809	0.9588	0.0148	0.0212	0.0101	0.0144	3
RF	0.9827	0.9569	0.0145	0.0218	0.0100	0.0153	4
DNN	0.9721	0.9023	0.0176	0.0321	0.0128	0.0243	5

a RMSE and MAE are in units of MPa^−1^. Values are means over 30 independent runs (*N* = 30). Models are ranked by test R^2^.

CatBoost ranks first with a test R^2^ of 0.9665, a training R^2^ of 0.9884, a test RMSE of 0.0190, and a test MAE of 0.0131. The gap between training and test R^2^ is 0.0219, the smallest among the five models, indicating that its ordered boosting and symmetric tree structure effectively control overfitting. XGBoost ranks second, with a test R^2^ of 0.9600, a test RMSE of 0.0209, and a test MAE of 0.0142. Its training R^2^ is 0.9884, with a gap of 0.0282 from the test R^2^, showing slightly weaker generalization than CatBoost but still robust. LightGBM ranks third, with a test R^2^ of 0.9588, a test RMSE of 0.0212, and a test MAE of 0.0144. Its training-test R^2^ gap is 0.0221, close to that of CatBoost, but overall accuracy is slightly lower. RF ranks fourth, with a test R^2^ of 0.9569, a test RMSE of 0.0218, and a test MAE of 0.0153. Although RF reduces variance by aggregating multiple decision trees, its prediction errors are marginally higher than those of the top three models. DNNs perform the weakest, with a test R^2^ of only 0.9023 and a training R^2^ of 0.9721; the training-test R^2^ gap is 0.0698, indicating significant overfitting under the condition of 454 samples, making DNN unsuitable as a base model for this task.

Overall, CatBoost, XGBoost, and LightGBM exhibit comparable performance with distinct characteristics, making them suitable as base models for stacking. RF is secondary, and DNN is excluded from subsequent ensembling.

To establish a baseline for the boosting-based models, traditional regression methods were evaluated under the same conditions. A multiple linear regression (MLR) using all nine physical indices, and univariate linear regressions using the liquidity index and water content, were fitted on the same training set and evaluated on the same test set. The results are summarized in Table 3. The MLR yields a test R^2^ of 0.9384, exceeding the DNN (R^2^ = 0.9023), while the univariate regressions perform substantially worse. All three boosting-based models, however, achieve test R^2^ values above 0.95, substantially exceeding both the linear and deep learning baselines. This pronounced performance gap indicates that the relationship between basic physical indices and compressibility in these soils cannot be reduced to linear additive functions, and motivates the ensemble approach developed in the following sections.

**Table 3:** Performance of traditional regression methods

Model	R^2^	RMSE^a^	MAE^a^
Multiple linear regression (9 inputs)	0.9384	0.0259	0.0201
Simple linear regression: a_v_ ∼ I_L_	0.8615	0.0390	0.0292
Simple linear regression: a_v_ ∼ w	0.7889	0.0480	0.0367

a RMSE and MAE are in units of MPa^−1^. All models were trained and evaluated on the same train-test splits as the base models in Table 2.

### Topology optimization and performance evaluation of the ensemble model

#### Meta-model selection

**Figure 3:**
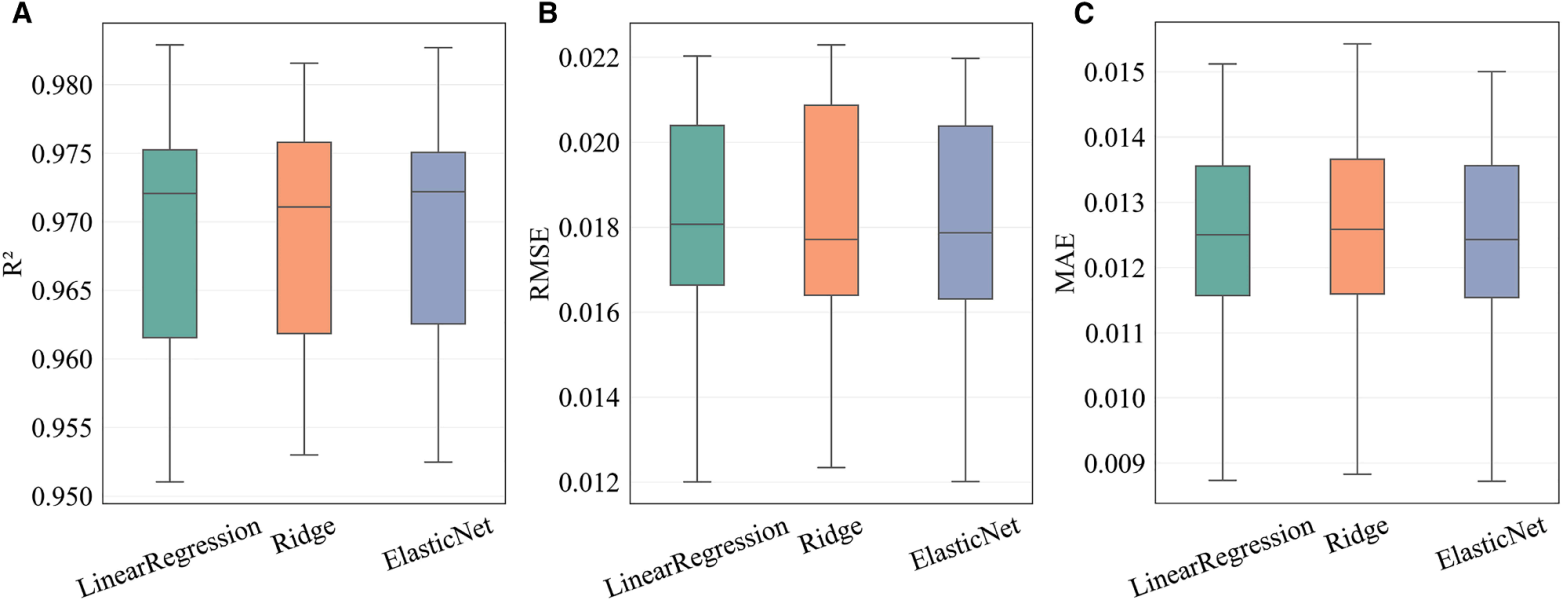
Boxplots of performance metrics for three meta-models on the test set over 30 independent runs (A) R^2^ distribution. (B) RMSE distribution (MPa^−1^). (C) MAE distribution (MPa^−1^). Boxes represent the interquartile range, horizontal lines indicate the median, and whiskers extend to 1.5 × IQR. Data are based on 30 independent train-test splits (*N* = 30). See also Table 4.

To determine the optimal meta-model configuration in the stacking framework, linear regression, ridge regression, and elastic net are compared as meta-models, with CatBoost, XGBoost, and LightGBM optimized by BOHB as base models. Table 4 summarizes the mean R^2^, RMSE, and MAE of each meta-model on the training and test sets. Figure 3 presents the 30-run distributions of these metrics on the test set using boxplots.

**Table 4:** Evaluation of meta-models (30 independent training-test data splits)

Base model (BOHB opt)	Meta model (Grid search)	R^2^ (mean)		RMSE (mean)^a^		MAE (mean)^a^		Ranking (R^2^)
		Train	Test	Train	Test	Train	Test	
BOHB-CatBoost-XGBoost-LightGBM	Linear regression	0.9708	0.9690	0.0188	0.0183	0.0130	0.0130	2
	Ridge	0.9699	0.9689	0.0191	0.0184	0.0132	0.0126	3
	ElasticNet	0.9706	0.9692	0.0189	0.0183	0.0130	0.0125	1

a RMSE and MAE are in units of MPa^−1^. Values are means over 30 independent runs (*N* = 30).

ElasticNet performs best, achieving the highest test R^2^ (0.9692) and the lowest test RMSE (0.0182) and MAE (0.01251), ranking first. Its training R^2^ is 0.9706, with a gap of only 0.0014 from the test R^2^, indicating the most robust generalization. This advantage is attributed to ElasticNet combining L1 and L2 regularization, which effectively screens redundant information among meta-features and controls coefficient inflation. Ridge regression ranks second, with a test R^2^ of 0.9689, a test RMSE of 0.0184, and a test MAE of 0.01263. Its training R^2^ is 0.9699, with a gap of 0.0010 from the test R^2^; although the generalization gap is small, its RMSE and MAE are slightly higher than those of ElasticNet. Linear regression ranks third, with a test R^2^ of 0.9690, a test RMSE of 0.0183, and a test MAE of 0.01257; its training R^2^ is 0.9708, and the training-test R^2^ gap is 0.0018. Although linear regression yields a lower RMSE than ridge regression, the boxplots in Figure 3 show that its R^2^ distribution across the 30 runs is wider, indicating higher sensitivity to data partitioning than ElasticNet; the variability of ridge regression is also slightly higher than that of ElasticNet. Therefore, considering both accuracy and stability, ElasticNet is the optimal meta-model. This study selects ElasticNet as the meta-model for the stacking framework.

#### Ensemble technique: stacking model based on CatBoost, XGBoost, LightGBM and BOHB

**Figure 4:**
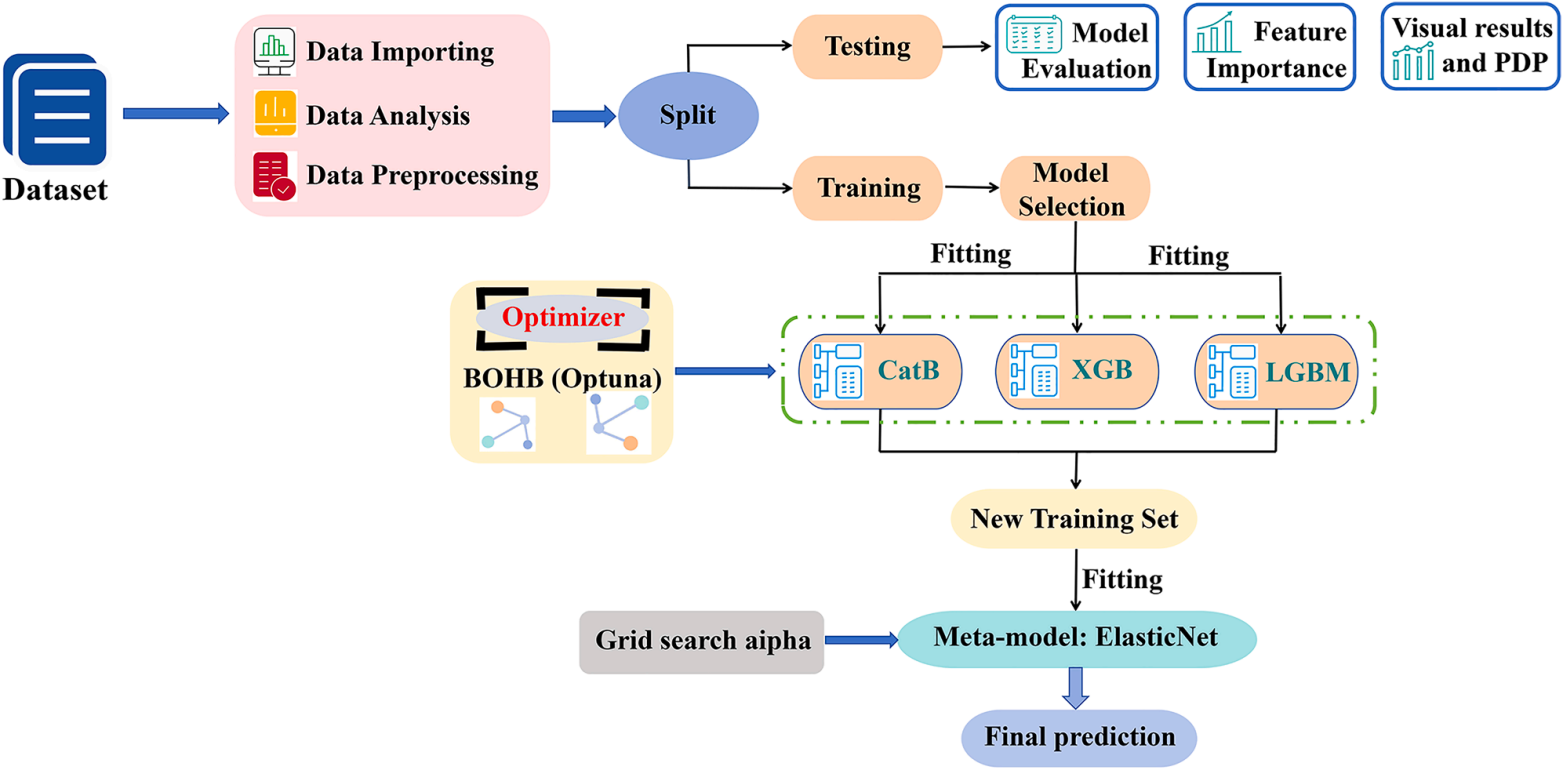
Workflow diagram of the stacking model Schematic illustration of the three-stage architecture: data preprocessing with *Z* score standardization and 85%/15% train-test split; base model training with three BOHB-optimized learners (CatBoost, XGBoost, LightGBM) using 5-fold cross-validation to generate meta-features; and meta-model training with ElasticNet to produce final predictions.

Using CatBoost, XGBoost, and LightGBM as base learners and ElasticNet as the meta-learner, this study constructs a stacking ensemble model. Figure 4 illustrates the specific workflow of this ensemble scheme. The data are randomly split into training and test sets at a ratio of 85%/15%, and 30 independent runs are repeated to evaluate the stability of the split. Model performance is assessed using R^2^, RMSE, and MAE. Within the training set, 5-fold cross-validation is employed to generate meta-features.

This framework combines the complementary decision boundaries of CatBoost, XGBoost, and LightGBM, while exploiting the sparsity of ElasticNet to enhance generalization stability.

Table 5 summarizes the hyperparameter configurations of each base model after BOHB optimization, along with the grid search results for the meta-model. The search ranges are based on preliminary experiments and previous studies,^16^^,^^17^ aiming to balance model capacity and generalization.

**Table 5:** Optimization details of the stacking model (CatBoost-XGBoost-LightGBM-ElasticNet)

Model	Hyper-parameter	Type	Range	Optimization
XGBoost	n_estimators	integer	[120, 480]	480
	max_depth	integer	[2, 5]	4
	learning_rate	float	[0.02, 0.2]	0.195
	subsample	float	[0.65, 0.95]	0.791
	colsample_bytree	float	[0.65, 0.95]	0.722
	min_child_weight	float	[1.0, 16.0]	1.864
	reg_lambda	float	[0.1, 20.0]	0.1
	reg_alpha	float	[1e−4, 5.0]	0.0001
	gamma	float	[0.0, 2.0]	0.0
CatBoost	iterations	integer	[200, 650]	565
	depth	integer	[3, 6]	3
	learning_rate	float	[0.02, 0.2]	0.0855
	l2_leaf_reg	float	[3.0, 15.0]	3.997
	subsample	float	[0.7, 1.0]	0.840
LightGBM	n_estimators	integer	[120, 500]	276
	max_depth	integer	[3, 7]	4
	learning_rate	float	[0.02, 0.2]	0.0452
	num_leaves	integer	[12, 40]	30
	subsample	float	[0.65, 0.95]	0.668
	colsample_bytree	float	[0.65, 0.95]	0.889
	min_child_samples	integer	[10, 40]	10
	reg_lambda	float	[0.1, 15.0]	0.1
	reg_alpha	float	[1e−4, 2.0]	0.0001
ElasticNet	alpha_1 (L1)	float	[0.01, 0.1, 1.0]	0.01
	alpha_2 (L2)	float	[0.01, 0.1, 1.0]	0.1
	l1_ratio	float	[0.5]	0.5

All base models were optimized using BOHB; the meta-model was tuned via grid search.

For XGBoost, n_estimators, max_depth, and learning_rate control model complexity: A lower learning rate requires more iterations but improves generalization; subsample and colsample_bytree introduce randomness to reduce variance; reg_lambda and reg_alpha provide L2/L1 regularization. For CatBoost, iterations, depth, and learning_rate determine the overall structural complexity; l2_leaf_reg applies L2 regularization to leaf weights; subsample controls the sampling ratio per iteration. For LightGBM, num_leaves is a key parameter controlling tree complexity, working together with max_depth to limit overfitting; min_child_samples enforces a minimum number of samples per leaf; reg_lambda and reg_alpha also provide regularization. For the meta-model ElasticNet, alpha_1 (L1) and alpha_2 (L2) are determined via grid search, with l1_ratio = 0.5 indicating an equal mixture of L1 and L2 penalties. Under the condition that the base model predictions may exhibit mild multicollinearity, this configuration improves the stability of the final prediction through sparse coefficient selection.

#### Performance evaluation of the optimized ensemble model

After determining the optimal ensemble topology, the prediction accuracy and split stability of the final model for a_v_ are evaluated through 30 independent runs. In each run, the dataset is randomly split into 85% training and 15% test sets (random seeds 1 to 30).

**Figure 5:**
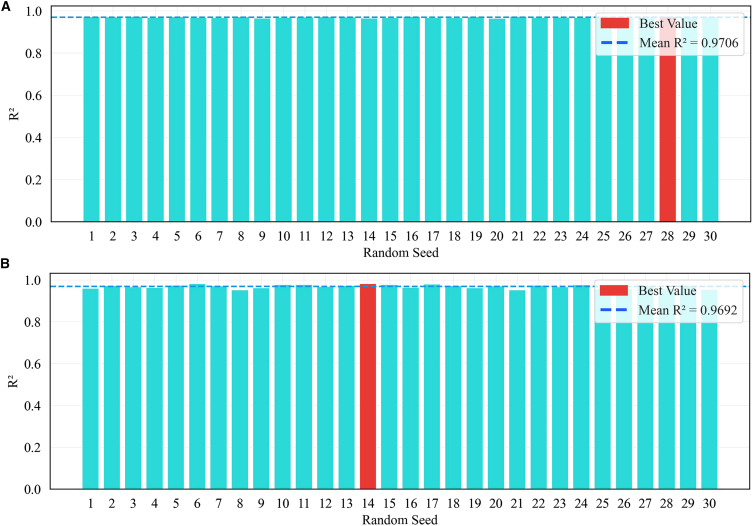
Stability of R^2^ values under different random seeds (A) Training set. (B) Test set. Each point represents one of the 30 independent runs (*N* = 30).

Figure 5 shows the variation of R^2^ on the training and test sets across the 30 runs. As shown in Figure 5A, the training R^2^ remains near 0.9706 throughout all runs, with almost no fluctuation, indicating that the model stably learns the patterns in the training data and that the data split has negligible impact on training performance. In contrast, Figure 5B reveals some variability in the test R^2^, with a mean of approximately 0.9692 and fluctuations within 0.002. Among the runs, random seed 28 achieves the highest test R^2^ (0.9698, highlighted in red), while seed 14 yields a relatively lower test R^2^ (0.9685). Despite this variability, the overall generalization capability remains highly consistent, demonstrating that the model has low sensitivity to random splitting.

**Figure 6:**
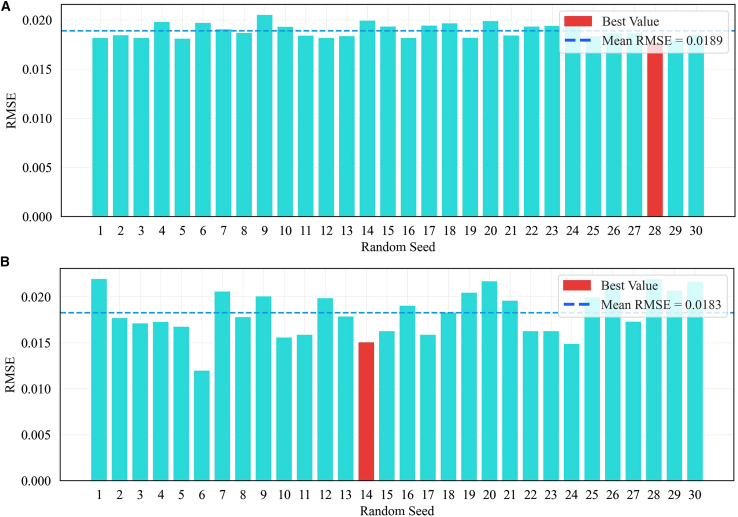
Stability of RMSE values under different random seeds (A) Training set. (B) Test set. RMSE is in units of MPa^−1^. Each point represents one of the 30 independent runs (*N* = 30).

Figure 6 evaluates the stability of RMSE. The training RMSE (Figure 6A) has a mean of approximately 0.0189, and the results across runs almost completely overlap, indicating that the model’s fitting error on the training set is highly stable. The test RMSE (Figure 6B) has a mean of 0.0183, with a slightly larger fluctuation range than that of the training set; the best run (random seed 14) achieves a test RMSE of 0.0150, while the worst run yields approximately 0.0215. Notably, the fluctuation range of test RMSE is substantially smaller than that of test R^2^, suggesting that the error metric is less sensitive to data partitioning than the coefficient of determination.

**Figure 7:**
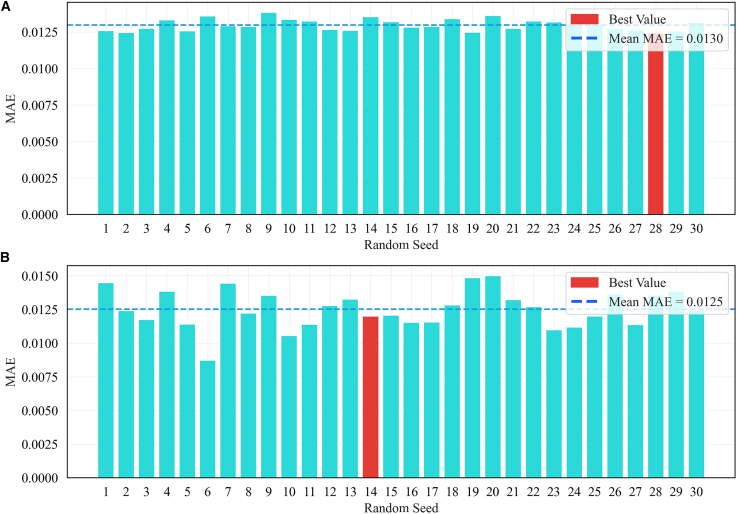
Stability of MAE values under different random seeds (A) Training set. (B) Test set. MAE is in units of MPa^−1^. Each point represents one of the 30 independent runs (*N* = 30).

Figure 7 evaluates the stability of MAE. The training MAE (Figure 7A) has a mean of 0.0130, with almost no difference across runs. The test MAE (Figure 7B) has a mean of 0.0125; the best run (random seed 14) achieves a test MAE of 0.0120, while the worst run yields approximately 0.0145. Combining the results from Figures 6 and 7, the stacking model exhibits excellent prediction stability across 30 different random splits, which is mainly attributed to the regularization mechanism of the ElasticNet meta-model and the improvement in base model generalization achieved by BOHB optimization.

**Figure 8:**
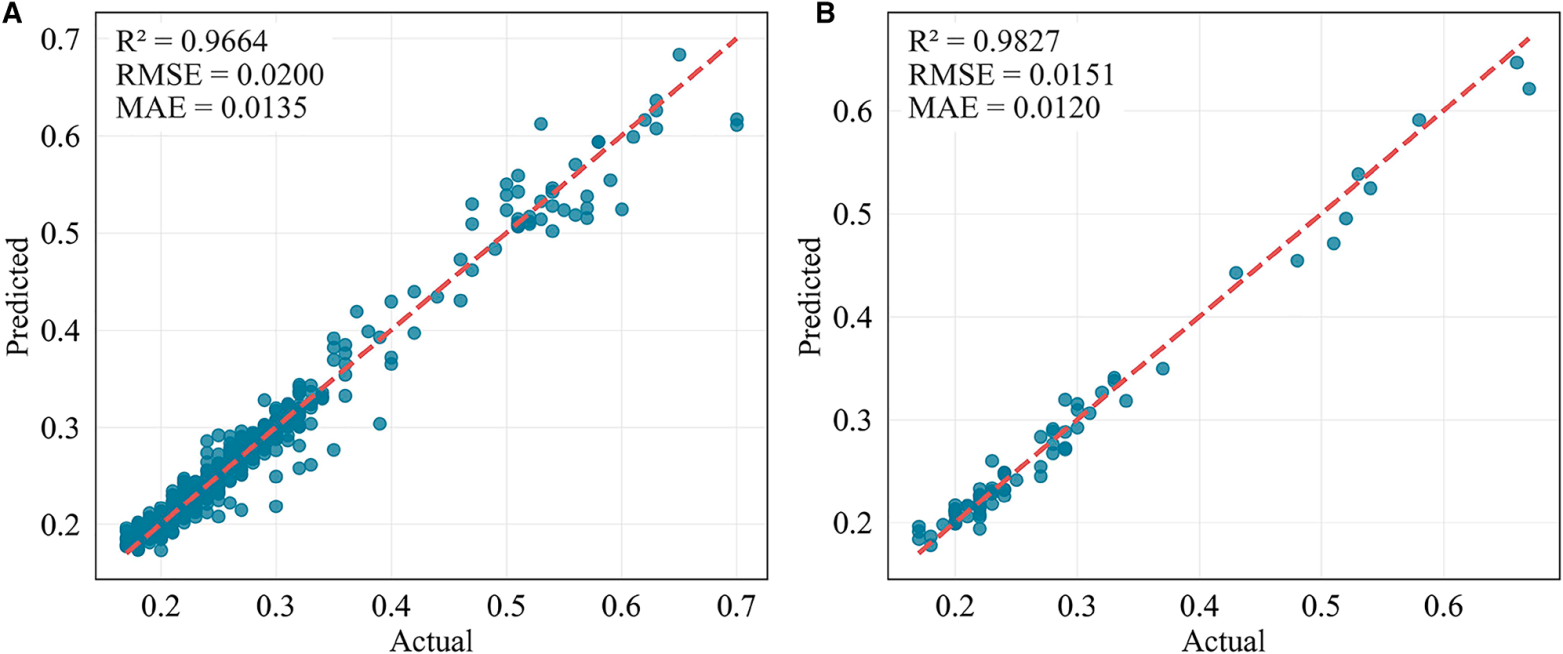
Regression plot of the stacking model (CatBoost-XGBoost-LightGBM-ElasticNet) for a representative run (seed 14) (A) Training set. (B) Test set. R^2^, RMSE, and MAE are displayed on each panel. Training set: *n* = 386; test set: *n* = 68.

To further visually assess prediction accuracy and error distribution, one representative run (random seed 14) is selected to generate regression and residual plots for both training and test sets. Figure 8A shows the relationship between predicted and true values on the training set: The data points are tightly distributed around the diagonal line, with R^2^ reaching 0.9664, RMSE of 0.0200 MPa^−1^, and MAE of 0.0135 MPa^−1^, indicating that the model has a strong fitting capability for the training data. The test set regression plot in Figure 8B shows that the model maintains excellent predictive performance on unseen data, with R^2^ increasing to 0.9827, RMSE of 0.0151 MPa^−1^, and MAE of 0.0120 MPa^−1^; the scatter points are uniformly distributed along the diagonal, with only a few samples showing slight deviations. The close similarity between the training and test error patterns, combined with the absence of systematic deviation in both regression plots, indicates that the model does not suffer from significant overfitting.

**Figure 9:**
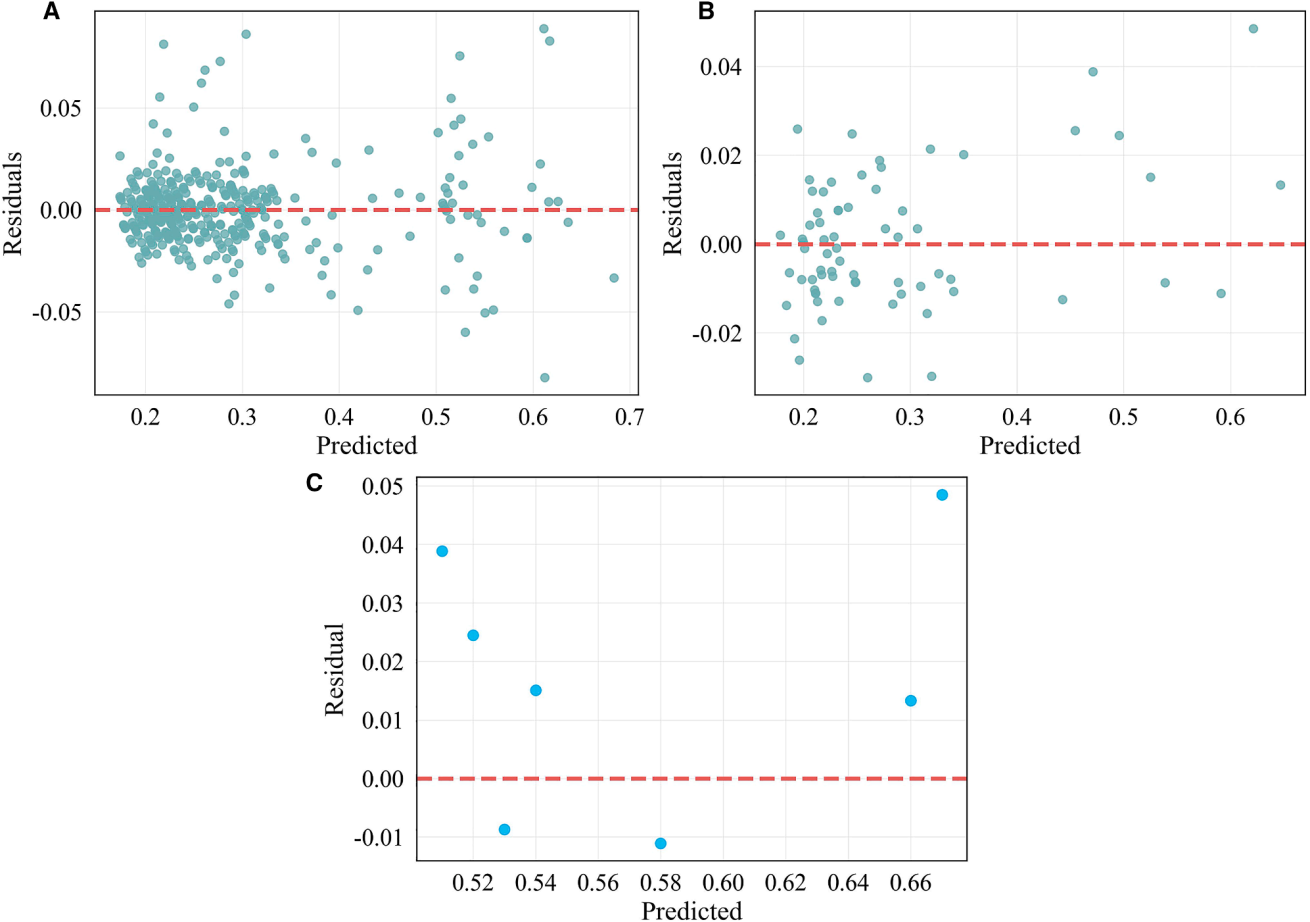
Residual plot of the stacking model (CatBoost-XGBoost-LightGBM-ElasticNet) (A) Training set. (B) Test set. (C) High-compressibility samples (a_v_ > 0.5 MPa^−1^). Residuals (MPa^−1^) are plotted against predicted values. The horizontal dashed line at zero represents perfect prediction. Training set: *n* = 386; test set: *n* = 68.

Figure 9 further reveals the error distribution characteristics. The training residual plot (Figure 9A) shows that the vast majority of residual points concentrate near the zero line, with absolute residuals mostly within 0.025 MPa^−1^; only a few extreme points extend to ±0.10 MPa^−1^, and no systematic patterns such as funnel shapes or curved trends are observed, indicating that the model fits the training data adequately without bias. The test residual plot (Figure 9B) exhibits an even narrower residual band, with absolute residuals of most samples within 0.02 MPa^−1^ and only a few samples approaching 0.04 MPa^−1^. The residuals are roughly symmetric around the zero line, with no significant heteroscedasticity, demonstrating that the model maintains consistent predictive reliability across the entire range of the coefficient of compressibility.

Notably, the samples with larger residuals in the test set mostly correspond to the high-compressibility region (true values > 0.5 MPa^−1^). These samples constitute a small proportion of the dataset—a reflection of the natural scarcity of highly compressible lateritic clays in the Guilin area, as evidenced by the pronounced right-skewness of the target variable (skewness = 1.74, Table 1). Given the engineering criticality of such extreme cases, a focused error analysis is performed on the high-compressibility samples from a representative test split (random seed 14). For this subgroup, the model achieves an RMSE of 0.0269 MPa^−1^ and an MAE of 0.0229 MPa^−1^. The residual plot for the high-compressibility samples (Figure 9C) shows that the largest deviation (0.0485 MPa^−1^) occurs for the sample with a true a_v_ of 0.67 MPa^−1^, where the model underpredicts by approximately 7.2%; the remaining residuals fall within ±0.04 MPa^−1^. Although the limited number of high-compressibility samples in the dataset precludes robust statistical generalization, the model consistently captures the order-of-magnitude and directional trend of these critical cases. For engineering practice, this level of accuracy suffices for preliminary site screening; nevertheless, when the model is deployed at locations where highly compressible lateritic clays are anticipated, local calibration using a modest number of oedometer tests is recommended prior to reliance on predictions for settlement-sensitive designs.

**Figure 10:**
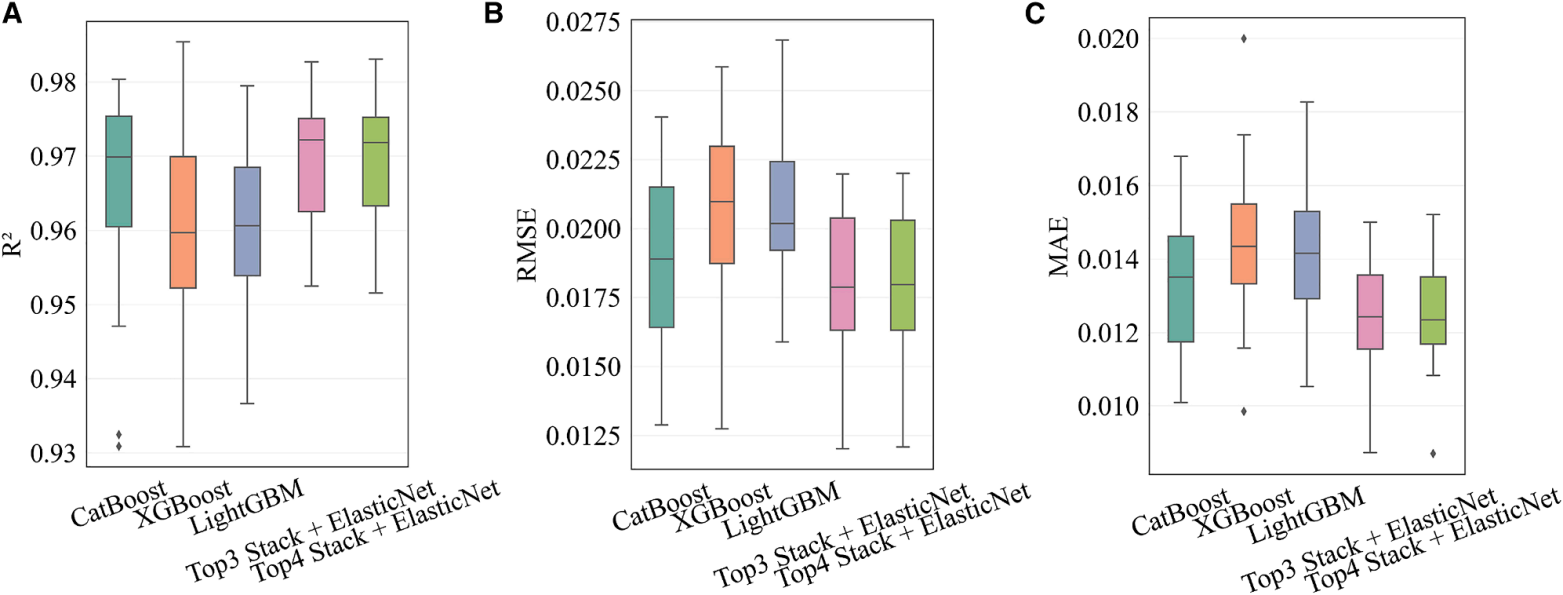
Boxplots of model performance over 30 independent runs (A) R^2^ distribution. (B) RMSE distribution (MPa^−1^). (C) MAE distribution (MPa^−1^). Boxes represent the interquartile range, horizontal lines indicate the median, and whiskers extend to 1.5 × IQR. Data are based on 30 independent runs (*N* = 30).

Table 6 summarizes the average performance of each model over 30 independent runs, and Figure 10 presents boxplots for a direct visual comparison of the distributions of each metric on the test set. Among the single base models, CatBoost performs best (test R^2^ = 0.9665, RMSE = 0.0190, MAE = 0.0131), with XGBoost and LightGBM ranking second and third, respectively. The three-model stacking ensemble optimized by BOHB (CatB-XGB-LGBM) raises the test R^2^ to 0.9692, reduces RMSE to 0.0183, and lowers MAE to 0.0125, significantly outperforming any single base model. The four-model stacking ensemble that further includes RF (CatB-XGB-LGBM-RF) achieves a slightly higher test R^2^ (0.9693) and a lower RMSE (0.0182), but the improvement is extremely marginal.

**Table 6:** Evaluation of stacking models (30 independent training-test data splits)

Model	R^2^ (mean)		RMSE (mean)^a^		MAE (mean)^a^		Ranking (R^2^)
	Train	Test	Train	Test	Train	Test	
CatB	0.9884	0.9665	0.0116	0.0190	0.0083	0.0131	3
XGB	0.9883	0.9600	0.0117	0.0209	0.0079	0.0142	4
LGBM	0.9809	0.9588	0.0148	0.0212	0.0101	0.0144	5
CatB-XGB-LGBM (opt)^b^	0.9706	0.9692	0.0189	0.0183	0.0130	0.0125	2
CatB-XGB-LGBM-RF (opt)^b^	0.9706	0.9693	0.0189	0.0182	0.0130	0.0125	1

a RMSE and MAE are in units of MPa^−1^. Values are means over 30 independent runs (*N* = 30).

b CatB, CatBoost; XGB, XGBoost; LGBM, LightGBM; RF, random forest. (opt) denotes BOHB-optimized.

To determine whether the performance difference between the three-model and four-model stacking ensembles is statistically significant, the Wilcoxon signed-rank test (α = 0.05) is performed on the R^2^, RMSE, and MAE results from the 30 independent runs. The test results are shown in Table 7. For all three metrics, the differences between the two models do not reach statistical significance (*p* > 0.05). Combined with the almost complete overlap of the performance distributions of the two stacking models in the boxplots (Figure 10), it can be concluded that the four-model stacking does not bring substantial improvement. Considering the computational cost of adding an additional base model, the three-model stacking achieves comparable prediction accuracy with higher computational efficiency. Therefore, this study selects the three-model stacking ensemble (CatBoost-XGBoost-LightGBM-ElasticNet) as the final prediction model.

**Table 7:** Wilcoxon signed-rank test results for performance differences between three-model and four-model stacking

Metric	Statistic	*P* value	Mean of three models	Mean of four models	Significant (α = 0.05)
R^2^	196	0.4645	0.9692	0.9693	No
RMSE^a^	191	0.4045	0.0183	0.0182	No
MAE^a^	221	0.8236	0.0125	0.0125	No

a RMSE and MAE are in units of MPa^−1^. Data are based on 30 independent runs (*N* = 30). No significant differences were found for any metric (*p* > 0.05).

Compared with the best single base model, CatBoost, this model achieves an R^2^ improvement of 0.0031 (approximately 3.2% relative increase), an RMSE reduction of 3.8%, and an MAE reduction of 4.4%. To verify that these improvements are statistically robust rather than artifacts of particular data splits, a paired Wilcoxon signed-rank test was performed on the 30-run test metrics of the stacking model and CatBoost. The results, summarized in Table 8, confirm that the gains in R^2^, RMSE, and MAE are all statistically significant (*p* < 0.05). The model also exhibits excellent stability under 30 different data splits, and residual analysis reveals no systematic bias, indicating strong potential for engineering application.

**Table 8:** Wilcoxon signed-rank test results for performance difference between the stacking model and CatBoost

Metric	Statistic	*P* value	Mean of three models	CatBoost	Significant (α = 0.05)
R^2^	131	0.0364	0.9692	0.9665	True
RMSE^a^	132	0.0384	0.0183	0.0190	True
MAE^a^	121	0.0208	0.0125	0.0131	True

a RMSE and MAE are in units of MPa^−1^. Data are based on 30 independent runs (*N* = 30). All differences are statistically significant (*p* < 0.05).

### Sensitivity analysis based on feature importance methods

**Figure 11:**
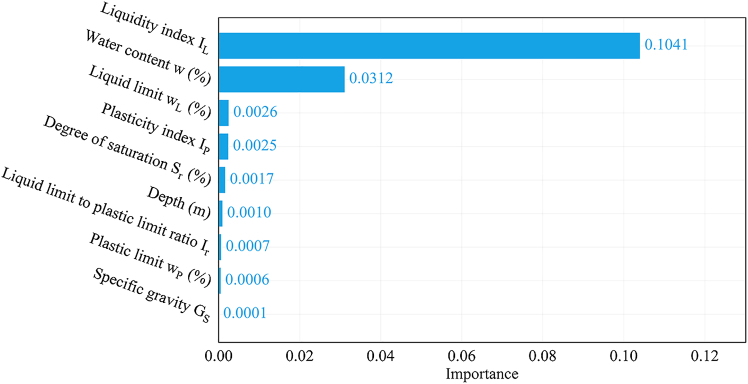
Permutation importance of input variables Importance scores represent the decrease in R^2^ when each feature is randomly permuted. Data are based on 454 samples (*n* = 454).

**Figure 12:**
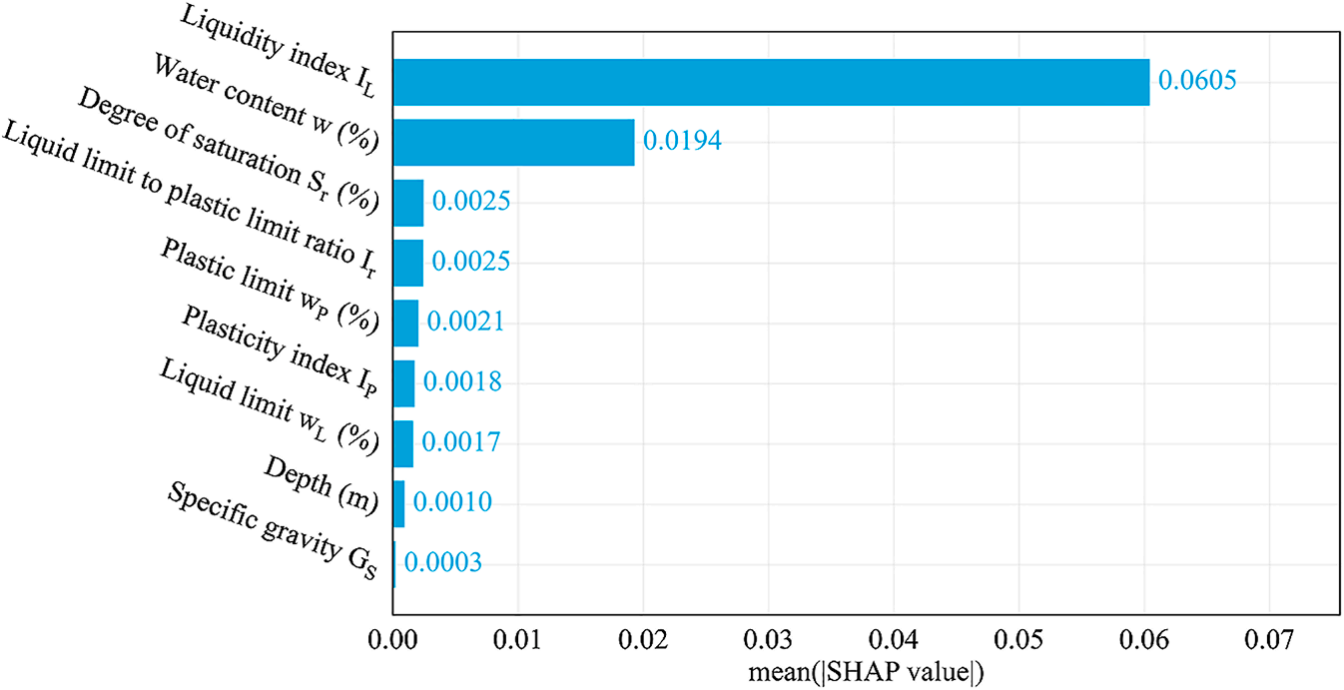
SHAP feature importance of input variables SHAP bar plot showing the mean absolute SHAP values for each input variable, quantifying each feature’s average contribution to the model output. Features are ranked by importance from top to bottom. Data are based on 454 samples (*n* = 454).

**Figure 13:**
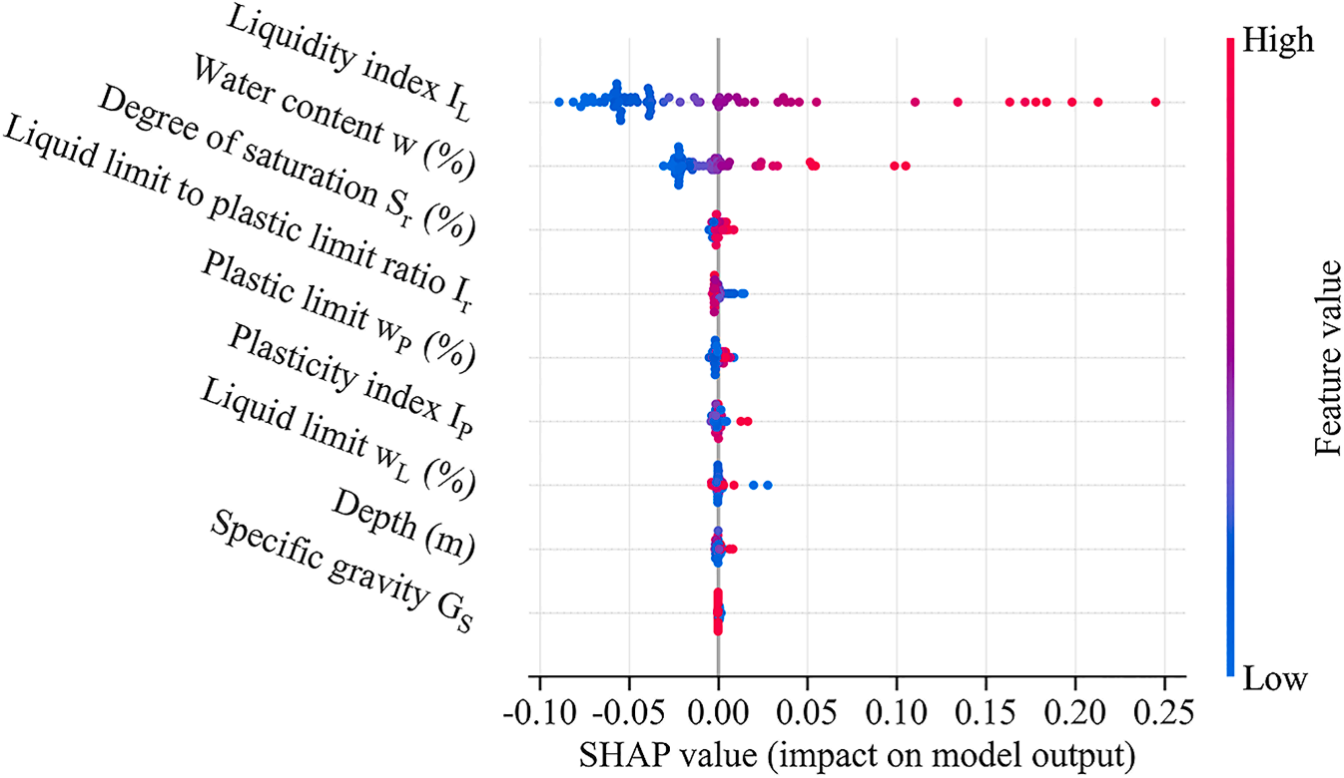
SHAP beeswarm plot of input variables SHAP summary plot showing the impact of each feature on model output. Each point represents one sample (*n* = 454), with color indicating feature value (red: high, blue: low). Positive SHAP values indicate a positive contribution to predicted a_v_. Features are ranked by importance from top to bottom.

To identify which physical indices most strongly influence the coefficient of compressibility, the relative importance of each input variable was quantified. SHAP analyses were performed on the final ensemble as a whole, attributing predictions directly to the nine original input features. Figure 11 presents the ranking of permutation importance. Figures 12 and 13 show the SHAP bar plot and the SHAP beeswarm plot, respectively.

Permutation importance (Figure 11) shows that the liquidity index (I_L_) ranks first with a score of 0.1041, and natural water content (w) ranks second with a score of 0.0312. The importance scores of the remaining variables are all substantially lower (≤0.0026). The SHAP bar plot (Figure 12) exhibits a consistent importance ranking: The liquidity index has the highest mean |SHAP| value (0.0605), followed by natural water content (0.0194), and the contributions of both are considerably higher than those of the remaining variables.

The SHAP beeswarm plot (Figure 13) further reveals the direction of influence and nonlinear characteristics. The SHAP values of the liquidity index increase markedly as the feature value increases, exhibiting a clear positive correlation trend, indicating that softer soil leads to a larger coefficient of compressibility. Natural water content similarly shows a monotonically increasing positive effect. The SHAP values of saturation (Sr), liquid-plastic ratio (Ir), plastic limit (w_P_), plasticity index (I_P_), liquid limit (w_L_), depth (D), and specific gravity (Gs) are all concentrated near zero with narrow distributions, indicating very limited influence on the model output. Among these, specific gravity contributes the least.

I_L_ and w are strongly correlated (r > 0.8, Figure 2), and this dependency means that the importance scores assigned to these two variables by permutation and SHAP reflect their joint contribution rather than independent effects of each. Accordingly, while both variables receive the highest importance across both methods, their individual ranking order does not necessarily indicate that one exerts a greater physical influence on compressibility than the other. Taken together, these results point to the combined influence of soil moisture and consistency state as the dominant factor governing the compressibility of Guilin lateritic clays. The remaining indices receive uniformly low importance across both methods, indicating that their direct predictive contribution is limited within the range of conditions covered by the dataset. This finding carries clear engineering implications: Settlement calculations should prioritize the consistency state and *in situ* moisture conditions of the soil. The low importance of indices such as plasticity index and depth reflects the unique microstructural characteristics of lateritic clays, where high clay content and cementation weaken the direct regulatory effect of these indices on compressibility.

### Partial dependence analysis

#### Accumulated local effects (ALE) and two-dimensional partial dependence analysis

**Figure 14:**
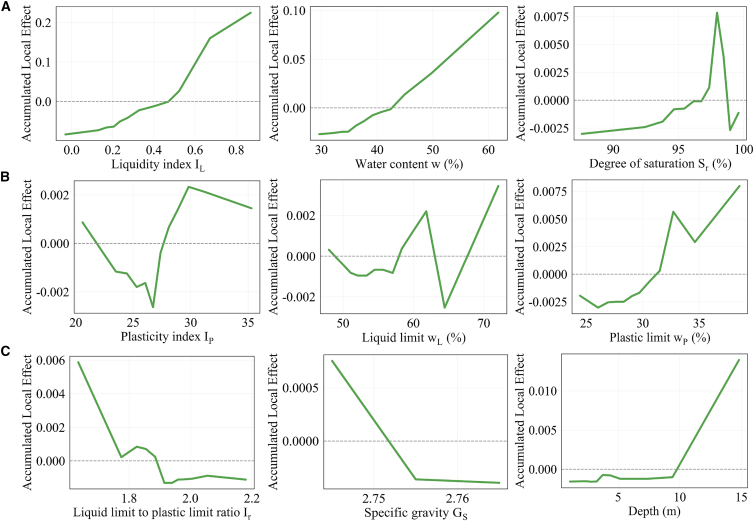
Accumulated local effects (ALEs) analysis (A) Liquidity index (I_L_), water content (w), and degree of saturation (Sr). (B) Plasticity index (I_P_), liquid limit (w_L_), and plastic limit (w_P_). (C) Liquid limit to plastic limit ratio (Ir), specific gravity (Gs), and depth (D). ALE curves show the cumulative local effect of each feature on predicted a_v_ (MPa^−1^). Data are based on 454 samples (*n* = 454).

Building on the global importance rankings established above, the functional relationship between each individual feature and the model predictions was examined to understand how these variables drive compressibility. Figure 14 shows the ALE curves of nine key input variables. The ALE curve of the liquidity index (I_L_) exhibits a pronounced monotonic positive correlation: When I_L_ increases from 0 to approximately 0.4, the ALE value rises slowly; beyond 0.4, the slope of the curve increases markedly, and the ALE value enters a phase of rapid growth, indicating that after the soil enters a soft plastic to fluid plastic state, compressibility increases sharply with the liquidity index. The ALE curve of natural water content (w) similarly shows a sustained positive cumulative effect. As water content increases from 30% to 60%, the ALE value turns from negative to positive and continues to rise, without an obvious saturation or plateau segment. Among the remaining variables, only the ALE curve of saturation (Sr) exhibits a local peak near the 100% range, while the ALE curves of plasticity index (I_P_), liquid limit (w_L_), plastic limit (w_P_), liquid-plastic ratio (Ir), specific gravity (Gs), and depth (D) show extremely small overall variations, with negligible independent driving effects on the coefficient of compressibility.

**Figure 15:**
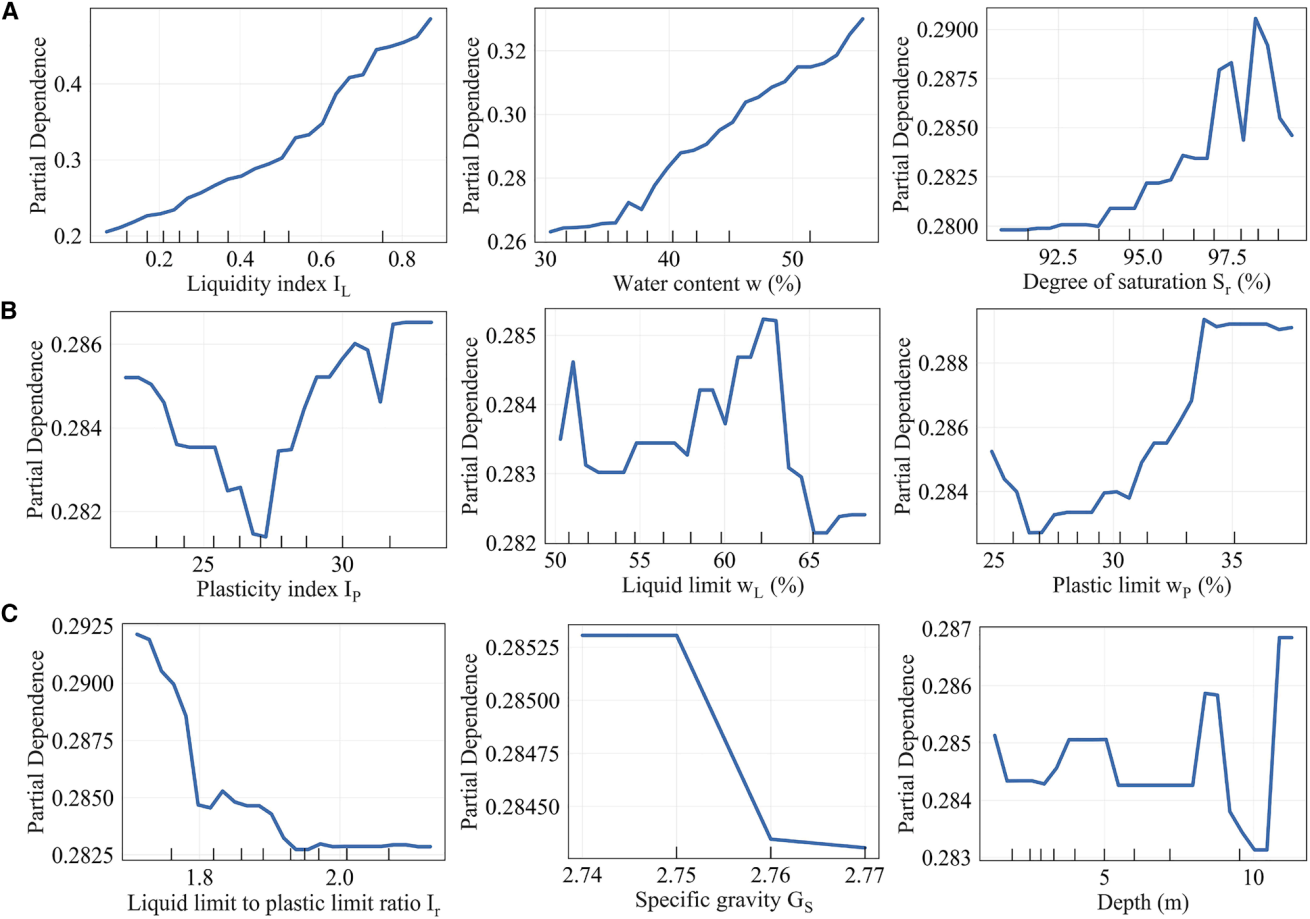
Univariate partial dependence plot (PDP) analysis (A) Liquidity index (I_L_), water content (w), and degree of saturation (Sr). (B) Plasticity index (I_P_), liquid limit (w_L_), and plastic limit (w_P_). (C) Liquid limit to plastic limit ratio (Ir), specific gravity (Gs), and depth (D). PDP curves show the marginal effect of each feature on predicted a_v_ (MPa^−1^). Data are based on 454 samples (*n* = 454).

Figure 15, showing univariate PDPs, further validates the above conclusions. The PDP curves of the liquidity index and natural water content are both monotonically increasing, corroborating the positive cumulative effects observed in the ALE analysis and confirming them as the two most influential core variables. The PDP curves of the liquid-plastic ratio and specific gravity exhibit slowly decreasing monotonic trends, but the overall magnitudes of change are very small. Although the PDP curves of plasticity index, liquid limit, plastic limit, saturation, and depth show local fluctuations, they lack significant monotonic trends, indicating limited independent influence.

Synthesizing the ALE and PDP results, the liquidity index and natural water content are the core driving factors controlling the coefficient of compressibility of lateritic clays. Their positive influence on model predictions continuously strengthens as their values increase. The independent influences of the remaining variables are generally weak, exhibiting only minor local fluctuations without playing a dominant role in the model output. This finding provides a clear basis for feature selection in predicting the coefficient of compressibility of lateritic clays and offers quantitative support for prioritizing key parameters and simplifying analyses in engineering practice.

#### 3D PDP

**Figure 16:**
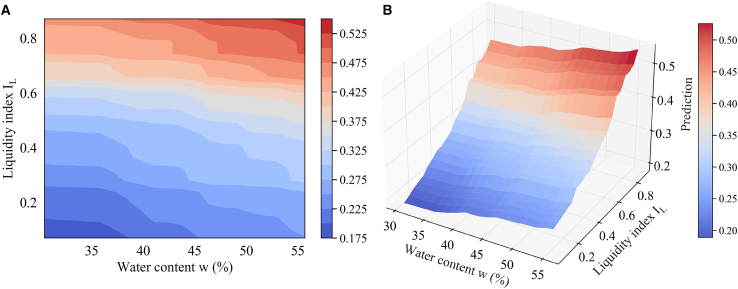
Interaction effects of water content and liquidity index on a_v_ (A) 2D contour plot. (B) 3D surface plot. Predicted a_v_ values are in units of MPa^−1^. Data are based on 454 samples (*n* = 454).

The univariate analyses above treat each feature in isolation. In practice, soil physical properties do not act independently—the effect of one index on compressibility may depend on the level of another. To capture such potential interactions, three-dimensional partial dependence plots and contour plots were constructed for four pairs of engineering-relevant features. Figure 16 presents the joint effect of the liquidity index (I_L_) and natural water content (w). The three-dimensional surface exhibits a pronounced “diagonal uplift” characteristic. When I_L_ < 0.2 and w < 35%, the coefficient of compressibility remains in the low range of 0.175–0.225 MPa^−1^. As the two variables increase simultaneously, the surface continuously rises along the diagonal direction. When I_L_ > 0.8 and w > 55%, the coefficient of compressibility reaches the high range of 0.475–0.525 MPa^−1^. The contour plot shows that the isolines are generally distributed along the diagonal of I_L_ and w, and the high-value region expands as both variables increase together, indicating a strong synergistic enhancement effect: A significant increase in the coefficient of compressibility requires both the liquidity index and natural water content to be in high ranges simultaneously; a change in a single variable alone is unlikely to trigger an abrupt change in compressibility. This result corroborates the positive cumulative effects of each variable observed in the ALE analysis while clearly revealing the nonlinear nature of their interaction.

**Figure 17:**
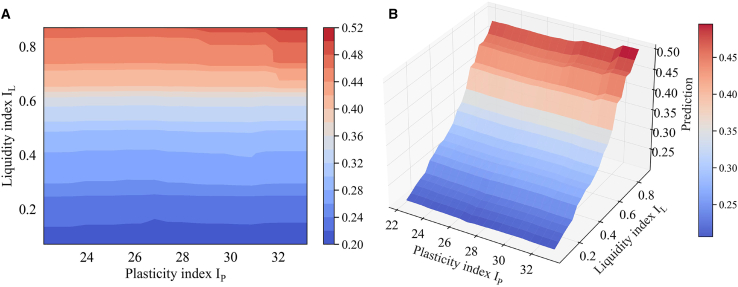
Interaction effects of liquidity index and plasticity index on a_v_ (A) 2D contour plot. (B) 3D surface plot. Predicted a_v_ values are in units of MPa^−1^. Data are based on 454 samples (*n* = 454).

Figure 17 presents the joint effect of the liquidity index (I_L_) and the plasticity index (I_P_) on the coefficient of compressibility. The variation of the three-dimensional surface is dominated by I_L_. When I_L_ < 0.2, the coefficient of compressibility remains stable in the range of 0.20–0.24 MPa^−1^, independent of I_P_. As I_L_ increases, the coefficient of compressibility rises continuously along the I_L_ axis, while the variation along the I_P_ axis is extremely limited. Only when I_L_ > 0.7 do highly plastic samples exhibit a slightly higher compressibility than low-to medium-plasticity samples, indicating that the plasticity index exerts only a weak facilitating effect under extreme liquidity conditions. The contour plot shows that the isolines are roughly parallel to the I_P_ axis, and the high-value region expands only with increasing I_L_, further confirming the above conclusion. This finding is consistent with the low importance of the plasticity index observed in the SHAP and ALE analyses.

**Figure 18:**
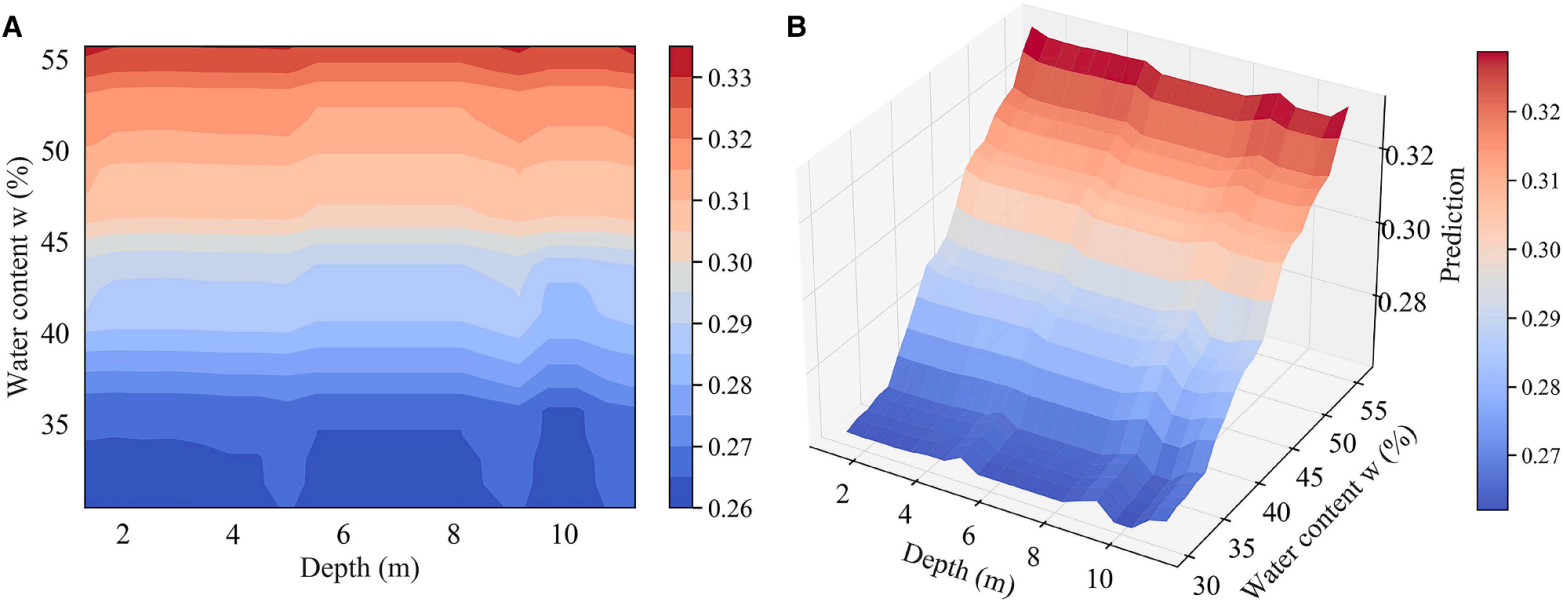
Interaction effects of water content and depth on a_v_ (A) 2D contour plot. (B) 3D surface plot. Predicted a_v_ values are in units of MPa^−1^. Data are based on 454 samples (*n* = 454).

Figure 18 presents the joint effect of natural water content (w) and sampling depth (D) on the coefficient of compressibility. The variation of the three-dimensional surface is dominated by water content. When w < 35%, regardless of depth, the coefficient of compressibility remains stable in the low range of 0.26–0.28 MPa^−1^. As water content increases, the surface continuously rises along the w-axis; when w > 55%, the coefficient of compressibility reaches the high range of 0.32–0.33 MPa^−1^. Along the depth axis, the surface is nearly horizontal, with a depth increase from 2 m to 10 m causing a change in the coefficient of compressibility of no more than 0.02 MPa^−1^. The contour plot shows that the isolines are roughly parallel to the depth axis, and the high-value region expands only with increasing water content, indicating no significant interaction between the two variables. This suggests that depth has a much smaller influence on compressibility than water content, and that settlement assessments in Guilin lateritic clay areas should prioritize the moisture condition of the soil over burial depth.

**Figure 19:**
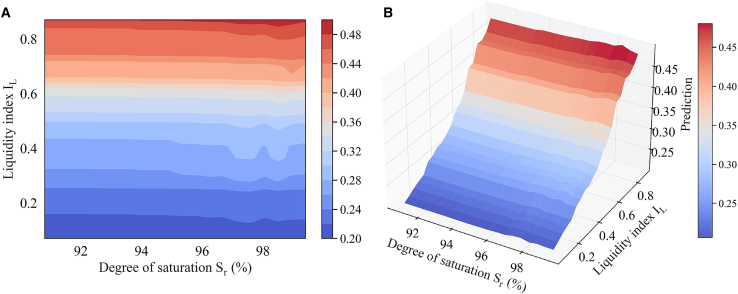
Interaction effects of liquidity index and degree of saturation on a_v_ (A) 2D contour plot. (B) 3D surface plot. Predicted a_v_ values are in units of MPa^−1^. Data are based on 454 samples (*n* = 454).

Figure 19 presents the joint effect of the liquidity index (I_L_) and saturation (Sr) on the coefficient of compressibility. The dominant variation of the three-dimensional surface is governed by the liquidity index. When I_L_ < 0.2, regardless of saturation, the coefficient of compressibility remains stable in the low range of 0.20–0.24 MPa^−1^. As I_L_ increases, the surface continuously rises along the I_L_-axis; when I_L_ > 0.8, the coefficient of compressibility reaches the high range of 0.44–0.50 MPa^−1^. Along the saturation axis, a change from 92% to 98% causes a variation in compressibility of no more than 0.04 MPa^−1^. Only in the high I_L_ range do samples with higher saturation exhibit a slightly higher compressibility than those with lower saturation. The contour plot shows that the isolines are roughly parallel to the saturation axis, and the high-value region expands only with increasing I_L_, indicating that saturation has a weak influence on compressibility, exerting only a mild synergistic enhancement under conditions of high liquidity index.

Synthesizing the above three-dimensional partial dependence analysis, the following patterns emerge from the model output. (1) When I_L_ is low (below approximately 0.2) and w is concurrently low (below approximately 35%), the predicted a_v_ remains in the low compressibility range, typically below 0.20 MPa^−1^. (2) The PDP surfaces further indicate that a_v_ rises into the high compressibility range (a_v_ > 0.50 MPa^−1^) when both I_L_ and w are elevated; this transition becomes apparent in the vicinity of I_L_ ≈ 0.8 and w ≈ 55%. I_L_ and w are strongly correlated (r > 0.8, Figure 2), and under such conditions PDP estimates can be biased toward unrealistic combinations of the two variables. The joint threshold values cited above therefore describe the modeled trend and remain approximate. They offer a preliminary empirical reference for identifying conditions under which high compressibility is more likely, and verification against independent site data is recommended before operational use. (3) The interaction analysis further demonstrates that a single variable alone is insufficient to drive a_v_ into the high compressibility range, underscoring the synergistic nature of the two factors.

### Prediction uncertainty analysis

The above analyses evaluate the model’s predictive accuracy and its physical interpretability. For engineering practice, equally important is the question of how stable these predictions remain when the input measurements fluctuate within the typical range of geotechnical testing variability. This was assessed by introducing random perturbations to the input features.

**Figure 20:**
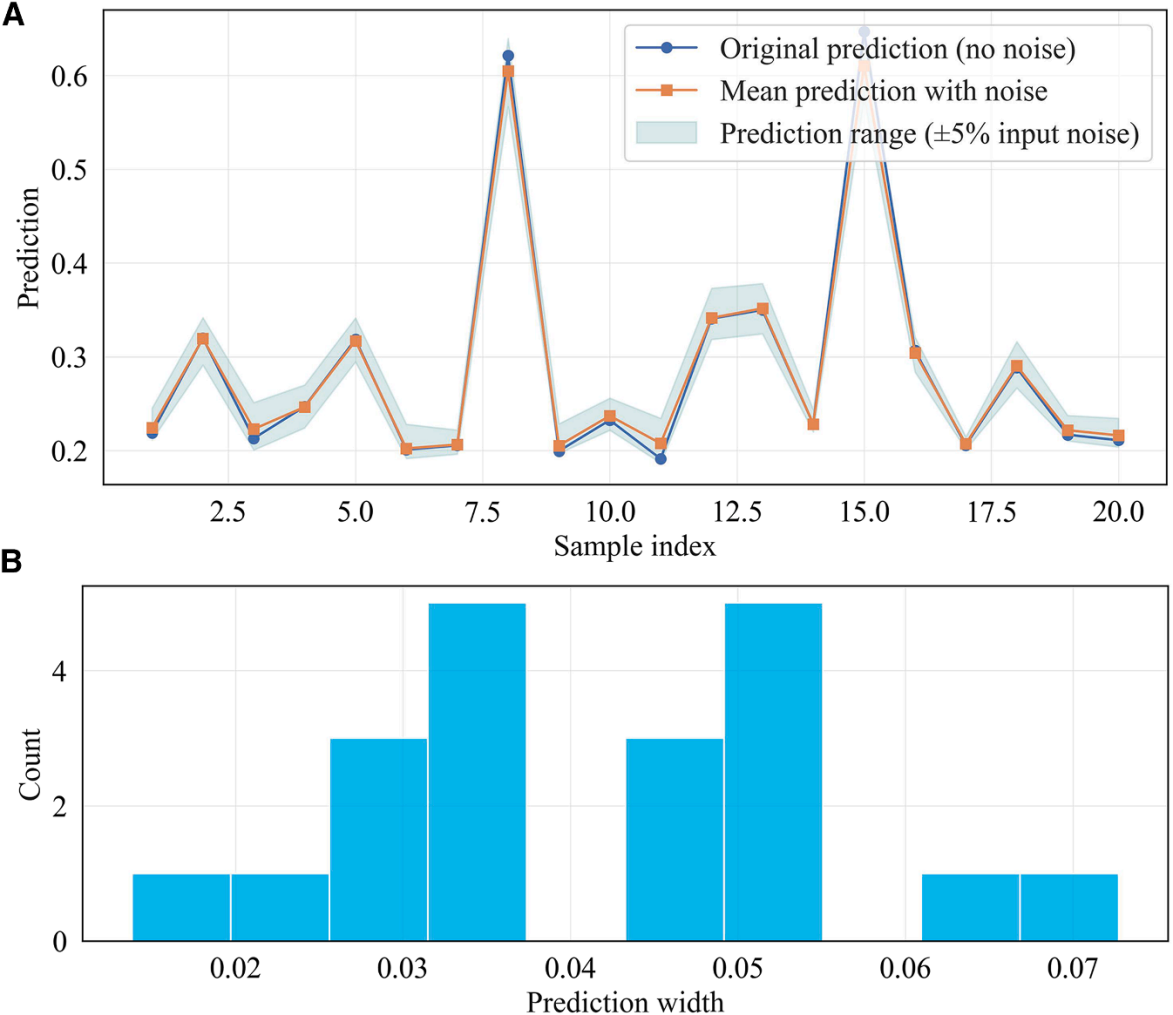
Prediction uncertainty under ±5% input noise from Monte Carlo simulation (A)Prediction uncertainty under ±5% input noise. (B) Histogram of prediction interval widths (MPa^−1^). Monte Carlo simulation was performed on 20 randomly selected test samples (*n* = 20) with 200 perturbed predictions per sample using Gaussian noise at ±5% of each feature value.

As shown in Figure 20A, the baseline predictions and noise-averaged predictions are highly consistent across samples, with nearly identical trends and minimal differences, indicating that the model maintains stable prediction direction under input perturbations. The 90% confidence intervals (shaded in gray) are generally narrow and uniformly distributed; only a few sample points (e.g., near indices 7.5–10.0 and 12.5–17.5) exhibit slight widening, but the magnitude remains controlled. The histogram in Figure 20B further quantifies the distribution of prediction width. The prediction widths are concentrated in the range of 0.02–0.06 MPa^−1^, with peaks near 0.03 MPa^−1^ and 0.05 MPa^−1^, and a mean width of approximately 0.04 MPa^−1^. About 80% of the samples have a prediction width below 0.05 MPa^−1^, and over 95% are below 0.07 MPa^−1^, indicating that under ±5% input noise, the dispersion of model predictions is relatively low, demonstrating excellent anti-interference capability.

Taken together, the Monte Carlo simulation results demonstrate that the model predictions remain stable under ±5% input perturbations, with 80% of samples exhibiting a prediction interval width below 0.05 MPa^−1^. This level of stability is particularly relevant for geotechnical engineering applications, where field and laboratory measurements are subject to inherent variability. It should be noted that the reported prediction intervals reflect only the contribution of input measurement noise to prediction variability; a complete quantification of predictive uncertainty would additionally require characterizing model form uncertainty and the natural spatial heterogeneity of lateritic clay properties.

### Model generalization ability evaluation

#### Spatial cross-validation

The preceding analyses have all been conducted under random data splitting, where samples from the same site may be distributed across both training and test sets. While this approach evaluates the model’s ability to interpolate within known geological conditions, it may provide an overly optimistic assessment of performance on entirely new sites—the scenario that matters most in engineering practice. To address this, a site-based cross-validation was carried out, in which all samples from a given site were held out together. Table 9 summarizes the R^2^, RMSE, and MAE on the training and test sets for each fold.

**Table 9:** Spatial cross-validation results (5-fold grouping)

Fold	Train			Test		
	R^2^	RMSE^a^	MAE^a^	R^2^	RMSE^a^	MAE^a^
1	0.9746	0.01863	0.01274	0.9005	0.02178	0.01549
2	0.9697	0.01992	0.01372	0.9766	0.01310	0.00983
3	0.9720	0.01688	0.01186	0.9692	0.02343	0.01607
4	0.9720	0.01897	0.01319	0.9565	0.01961	0.01494
5	0.9669	0.01851	0.01314	0.9045	0.04204	0.02179
Mean	0.9710	0.01858	0.01293	0.9415	0.02399	0.01562

a RMSE and MAE are in units of MPa^−1^. Spatial cross-validation (5-fold, grouped by investigation site so that all samples from the same site are held out together in each test fold).

As shown in Table 9, the test R^2^ fluctuates between 0.9005 and 0.9766, with a mean of 0.9415 and a standard deviation of 0.0324. Among the folds, Fold 2 performs best (test R^2^ = 0.9766, RMSE = 0.0131), while Fold 1 and Fold 5 perform relatively weaker (test R^2^ = 0.9005 and 0.9045, respectively), mainly due to the higher proportion of highly compressible samples in those sites. The mean test RMSE is 0.0240 MPa^−1^ with a standard deviation of 0.0105; the mean test MAE is 0.0156 MPa^−1^ with a standard deviation of 0.0045. The performance difference between training and test sets reflects the heterogeneity of soil properties across different sites. Some sites (e.g., those corresponding to Fold 1 and Fold 5) exhibit more complex soil characteristics or a more concentrated distribution of highly compressible samples (true values > 0.50 MPa^−1^). When these sites are assigned to the test set, the model’s prediction accuracy decreases correspondingly. The test R^2^ exceeded 0.90 for every fold, confirming that the model retains basic predictive capability even for the most challenging sites. The range from 0.9005 to 0.9766 across folds—and the systematic underperformance on folds with a high concentration of highly compressible samples—indicates that the model’s accuracy is site-dependent and that the average performance observed under random splitting (R^2^ = 0.9692) is likely to overestimate its predictive accuracy on sites whose soil characteristics differ substantially from those well represented in the training data.

#### Sample size sensitivity analysis

**Figure 21:**
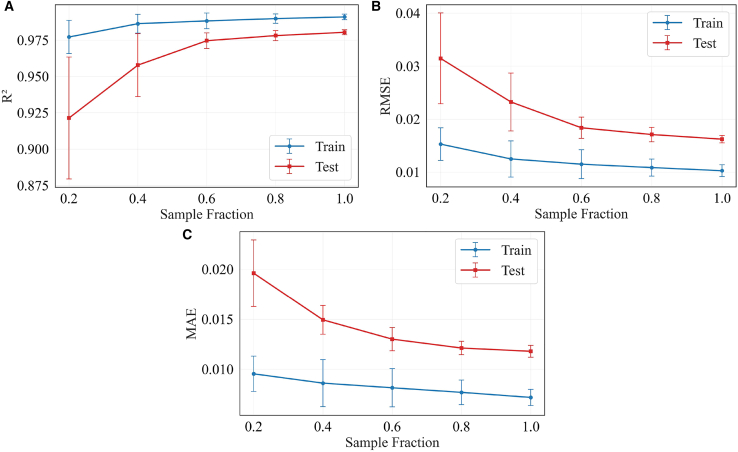
Sample size sensitivity analysis (A) R^2^. (B) RMSE (MPa^−1^). (C) MAE (MPa^−1^). Solid lines represent the mean over 10 repeated subsamples at each fraction (*N* = 10), with error bars indicating ±1 SD. Sample fractions shown on the x axis correspond to 20%, 40%, 60%, 80%, and 100% of the training set. The test set is fixed at 68 samples (15% of total).

The generalization assessments above were conducted with the full dataset of 454 samples. To guide future site investigation planning, it is useful to ask how many samples are actually needed to achieve a given level of predictive accuracy. This was addressed by tracking model performance as the training set was progressively reduced in size. Figure 21 shows the trend of each metric as the sample size increases.

As shown in Figure 21A, the test R^2^ exhibits a continuous upward trend as the sample size increases: When the sample size is 77 (20%), the mean test R^2^ is 0.921; when it increases to 154 (40%), it rises to 0.958; when it reaches 231 (60%), it further improves to 0.975; and when it reaches 385 (100% of the base training set), it further improves to 0.980. The training R^2^ remains consistently slightly higher than the test R^2^, but the gap between them narrows as the sample size increases—from 0.056 at 20% to 0.011 at 100%—indicating that overfitting is effectively mitigated with more data. Figures 21B and 21C show the opposite trend for RMSE and MAE: The test error continuously decreases as the sample size grows, with RMSE dropping from 0.0315 MPa^−1^ to 0.0162 MPa^−1^, and MAE from 0.0196 MPa^−1^ to 0.0118 MPa^−1^. The standard deviations of the metrics are relatively large when the sample size is small (e.g., at 20%, the standard deviation of test R^2^ is 0.042); as the sample size increases, the standard deviations gradually shrink, and at 100%, the standard deviation of test R^2^ falls to 0.002, indicating that the stability of model performance improves with more data.

These results suggest that the current sample size of 454 is largely sufficient for model training, and further increases in sample size would yield only marginal gains in performance. For the task of predicting the coefficient of compressibility of lateritic clays, approximately 230 training samples (60% of the base training set) already achieve a test R^2^ of 0.975, which is close to 99% of the final performance. This finding provides a reference for the rational planning of sample sizes in future regional site investigations.

## Discussion

### Performance advantages of the stacking model and its mechanisms

The BOHB-optimized stacking ensemble model (CatBoost-XGBoost-LightGBM-ElasticNet) achieves a test R^2^ of 0.9692, marginally higher than that of the best single base model CatBoost (0.9665). The improvement, while statistically significant (Table 8), is numerically modest—a gain of 0.0027 in R^2^ (a relative increase of approximately 2.8%) and reductions of 0.0007 MPa^−1^ (3.7%) in RMSE and 0.0006 MPa^−1^ (4.6%) in MAE. The training R^2^ of the stacking model (0.9706, Table 6) is lower than that of CatBoost (0.9884). This is intrinsic to the stacking procedure: Cross-validation in meta-feature generation introduces prediction error, and the L1/L2 regularization of the ElasticNet meta-model compresses coefficient degrees of freedom. Despite the lower training fit, the stacking model yields a lower test RMSE (0.01827 vs. 0.01900) and a higher test R^2^, indicating stronger generalization—a classic bias-variance trade-off in ensemble learning. The trade-off is consistent with the mean values reported in Table 6 and the significant test-set improvement shown in Table 8.

The practical value of this framework lies not in these marginal accuracy gains but in three other aspects, collectively establishing a full-chain intelligent prediction paradigm of “hybrid optimization—interpretable analysis—uncertainty quantification” and offering a reproducible technical pathway for intelligent prediction of regional geotechnical parameters. First, BOHB automates hyperparameter tuning, removing subjective manual choices and ensuring reproducible performance. Second, stacking reduces prediction variance by combining heterogeneous models, improving robustness against data variability—an essential requirement for geotechnical applications where soil properties exhibit inherent spatial heterogeneity (Table 1). Third, the interpretability tools (SHAP, ALE, PDP) provide qualitative insights into the physical drivers of compressibility, including the synergistic effect between I_L_ and w. The partial dependence analysis suggests approximate thresholds (I_L_ ≈ 0.8, w ≈ 55%) where a_v_ tends to rise into the high-compressibility range. These values should be viewed as exploratory indicators for preliminary site screening rather than as fixed design limits without further validation. These advantages originate from the complementarity of the base learners’ decision boundaries and the regularized screening of the meta-learner. CatBoost’s symmetric tree structure controls overfitting through ordered boosting; XGBoost captures nonlinear interactions via second-order gradient statistics; LightGBM, with its histogram-based algorithm, balances efficiency and accuracy. After BOHB optimization, all three reach their optimal configurations, and the ElasticNet meta-model screens effective predictive information through L1/L2 mixed regularization, avoiding overfitting from redundant base models.

The Wilcoxon test shows that the performance difference between the three-model stacking and the four-model stacking (including RF) is not statistically significant (*p* > 0.05), confirming that the current combination of base models is sufficient. This finding is corroborated by two recent independent studies in geotechnical machine learning. Van et al.^34^ found that the combination “XGBoost + CatBoost + Lasso” was optimal for predicting hydration heat in mass concrete, and Yuan et al.^35^ demonstrated through a systematic evaluation of 63 combinations derived from six base models that integrating 3–4 base models achieves optimal performance. These results suggest that on medium-sized datasets, three to four optimized heterogeneous base models generally suffice to capture the decision space, and blindly adding more base models may instead introduce redundant noise.

### Dominant role of liquidity index and water content and its physical mechanism

Consistent with soil mechanics principles, multiple model analyses consistently identify the liquidity index and natural water content as the two core factors controlling the coefficient of compressibility of lateritic clays, with their cumulative contribution exceeding 95%. From the perspective of soil mechanics, water content directly influences effective stress by regulating pore water pressure, while the liquidity index, as a comprehensive indicator of consistency state, is governed by the hydrophilicity of clay minerals.

SHAP analysis reveals a monotonic positive correlation for both variables, consistent with the principle of effective stress. The three-dimensional partial dependence analysis further uncovers a strong synergistic enhancement effect: The coefficient of compressibility tends to rise into the high range (>0.50 MPa^−1^) when I_L_ and w are jointly elevated—with the transition observed approximately at I_L_ ≈ 0.8 and w ≈ 55%—rather than being triggered by a high value of a single variable alone. While these numerical markers offer a useful empirical reference for identifying conditions conducive to high compressibility in Guilin lateritic clays, they should be viewed as exploratory guides for preliminary site screening, not as fixed design limits—site-specific oedometer tests remain indispensable for settlement-sensitive designs. The practical takeaway of this nonlinear interaction is that settlement prediction in lateritic clay areas must concurrently consider both the consistency state and the *in situ* moisture condition—neither factor can be neglected. By contrast, the plasticity index exerts only a weak promoting effect on compressibility under high liquidity conditions and contributes marginally to the overall prediction, indicating that the compressibility of lateritic clays is largely insensitive to plasticity.

### Site heterogeneity challenges revealed by spatial cross-validation

The spatial cross-validation results (mean test R^2^ = 0.9415, standard deviation = 0.0324) quantify the impact of site heterogeneity on model generalization. The test R^2^ values of Fold 1 (0.9005) and Fold 5 (0.9045) are markedly lower than that of Fold 2 (0.9766). This is because the highly compressible samples (>0.5 MPa^−1^) are mainly concentrated in the sites corresponding to Folds 1 and 5. When these sites are assigned to the test set, the model faces out-of-distribution predictions, and its accuracy naturally declines.

Zhan et al.^36^ showed that random cross-validation, by ignoring spatial correlations, overestimates model performance, whereas spatial cross-validation provides a more realistic benchmark of generalization capability. Kumar et al.^37^ also support this view, demonstrating that random cross-validation overestimates the performance of landslide susceptibility models and emphasizing the necessity of spatial validation in geotechnical machine learning. The present results are consistent with these findings: the test performance under random splitting (R^2^ = 0.9692) is higher than the mean spatial cross-validation R^2^ (0.9415), by approximately 2.9%, with the latter more faithfully reflecting the model’s predictive capability for unsurveyed sites. This more realistic assessment, however, varies considerably from site to site: the 2-folds with the lowest test R^2^ (Fold 1: 0.9005; Fold 5: 0.9045) contain the highest proportions of samples with a_v_ > 0.5 MPa^−1^—samples that are sparsely represented in the overall dataset (Table 1). When a site dominated by highly compressible lateritic clay is entirely withheld from training, the model is forced to extrapolate beyond the regions of the feature space that are well-supported by its training distribution. The resulting decline in accuracy, from approximately 0.97 under random splitting to approximately 0.90 in the worst spatial folds, represents a realistic performance bound for applications to new, dissimilar sites, revealing the performance boundary of the model when facing site conditions not well represented in the training data. In practical terms, when deploying the model at a previously unsurveyed site—particularly one where soft, high-compressibility clay is anticipated—a small number of site-specific undisturbed samples should be collected for oedometer testing. If the model predictions on these samples fall within the error range seen in Folds 2–4 (R^2^ ≈ 0.96–0.98), the model can be applied across the remainder of the site with reasonable confidence. If larger deviations are observed, which is more likely when the new site is dominated by highly compressible material, the local calibration data can be used to fine-tune the model before full-scale settlement assessment.

### Model robustness validated by Monte Carlo simulation

The Monte Carlo simulation (with ±5% input noise) shows that 80% of the samples have prediction widths below 0.05 MPa^−1^, indicating that the model has good resistance to measurement errors. This characteristic is particularly critical in geotechnical engineering, where field sampling and laboratory tests inevitably involve disturbance and errors; the model’s robustness directly affects its reliability in engineering applications. Yi et al.^38^ also validated the effectiveness of Monte Carlo simulation for assessing model stability in their study on uncertainty quantification of rock mechanical parameters. As the Monte Carlo analysis perturbs only the input features, the resulting prediction intervals provide a lower-bound estimate of total predictive uncertainty, capturing the component attributable to measurement variability.

It is worth noting that samples with larger residuals (>0.04 MPa^−1^) mostly correspond to the high-compressibility region (>0.5 MPa^−1^). The focused error analysis shows that even for these extreme cases, the model’s predictions deviate only modestly from the true values—well within the typical reproducibility range of oedometer tests on structured lateritic clays. This performance is non-trivial because the high-compressibility regime coincides with the strong synergistic enhancement zone where I_L_ and w are jointly elevated, with thresholds approximately at I_L_ ≈ 0.8 and w ≈ 55% (Figure 16), where a_v_ becomes acutely sensitive to small input variations. The model’s ability to capture this nonlinear interaction despite the natural scarcity of such samples (skewness = 1.74, Table 1) attests to its extrapolation capacity. Moreover, the slight conservative bias observed in this tail—tending toward underprediction—implies that settlement risks would not be underestimated. These samples account for only about 10% of the total, reflecting that the model’s ability to fit the tail distribution still has room for improvement. This limitation is not unique to the present model but is a common issue for most data-driven methods when faced with skewed sample distributions. In the future, Synthetic Minority Over-Sampling Technique (SMOTE)^39^ could be adopted to further improve the model’s ability to fit extreme samples.

### Data requirements revealed by sample size sensitivity analysis

The learning curve analysis indicates that the current 454 samples are largely sufficient for model training. When the training subset reaches approximately 230 samples (60% of the base training set, corresponding to about 300 total samples), the test R^2^ already attains 0.975, which is 99% of the final performance (0.980). The training-test R^2^ gap narrows from 0.056 at 20% of the training subset to 0.011 at the full base training set, confirming that overfitting is alleviated as more data become available.

This finding provides a quantitative reference for sample size planning in regional site investigations: Under similar geological conditions, about 300 samples can support high-precision prediction, effectively balancing investigation cost and model performance. Bouasria et al.^40^ observed in a machine learning-based spatial prediction study that once the sample size exceeded 300–500, further increases in sample size yielded only marginal gains in predictive accuracy. Their observation aligns well with our results, reinforcing the notion that a moderate sample size of around 300 is sufficient to achieve reliable predictions in similar data-driven geotechnical modeling tasks.

### Limitations of the study

Although this study has made preliminary progress in predicting the coefficient of compressibility of Guilin lateritic clays using a BOHB-optimized stacking ensemble, further breakthroughs are still needed in the following three dimensions. First, moving from a region-specific model to a cross-region transferable framework: By integrating domain adaptation or physics-guided generative models, a general compressibility map applicable to lateritic clays across South China could be constructed. Second, advancing from macro-index-driven statistical fitting to a macro-micro multiscale mechanism-coupled approach: introducing microstructural parameters (e.g., the degree of iron-aluminum cementation quantified by XRD or SEM) or embedding physical priors into physics-informed neural networks would upgrade the prediction from statistical approximation to mechanism-driven modeling. Third, shifting from passive imbalanced-data processing to active learning combined with physics-constrained generation: developing physics-guided generative adversarial networks and active sampling strategies would enable closed-loop optimization between data acquisition and model iteration, thereby enhancing the reliability of tail-risk quantification for highly compressible samples.

## Resource availability

### Lead contact

Requests for further information and resources should be directed to and will be fulfilled by the lead contact, Hanying Bai (baihylh@glut.edu.cn).

### Materials availability

This study did not generate new datasets.

### Data and code availability

• The data reported in this paper are publicly available at Mendeley Data: https://doi.org/10.17632/xw52sjf9c7.1.
• All original code is publicly available at Zenodo: https://doi.org/10.5281/zenodo.20424543.
• Any additional information required to reanalyze the data reported in this paper is available from the lead contact upon request.

## Acknowledgments

This work was supported by the Guangxi Science and Technology Program (Guike LT2600640017). The authors are sincerely grateful for this support.

## Author contributions

T.W.: conceptualization, methodology, software, formal analysis, and writing – original draft. J.L.: data curation, validation, and writing – reviewing and editing. H.B.: conceptualization, resources, supervision, project administration, funding acquisition, and writing – reviewing and editing. All authors read and approved the final manuscript.

## Declaration of interests

The authors declare no competing interests.

## STAR★Methods

### Key resources table

**Table:** 

REAGENT or RESOURCE	SOURCE	IDENTIFIER
**Deposited data**		

Guilin Lateritic Clay Dataset (454 samples)	Authors (This study)	Mendeley Data: https://doi.org/10.17632/xw52sjf9c7.1

**Software and algorithms**		

Python (v3.9)	Python Software Foundation	https://www.python.org/
Visual Studio Code	Microsoft	https://code.visualstudio.com/
Code to reproduce the results of this study	Authors (This study)	Zenodo: https://doi.org/10.5281/zenodo.20424543

### Method details

#### Data collection and preprocessing

The dataset used in this study originates from 18 engineering investigation projects in the Guilin area of Guangxi, yielding a total of 454 undisturbed lateritic clay samples. All laboratory tests strictly followed the Standard for Geotechnical Testing Method (GB/T 50123–2019).^41^ The measured indices include sampling depth (D), natural water content (w), specific gravity of soil particles (Gs), saturation (Sr), liquid limit (w_L_), plastic limit (w_P_), plasticity index (I_P_), liquidity index (I_L_), liquid-plastic ratio (Ir), and coefficient of compressibility (a_v_). The liquid-plastic ratio (Ir) is defined as the ratio of liquid limit to plastic limit, i.e., Ir = w_L_/w_P_. Descriptive statistical analyses, including summary statistics, distribution visualization, and correlation analysis, were first performed on the raw data. Subsequently, to prepare the data for machine learning modeling, *Z* score standardization was applied to all continuous variables, transforming each feature into a distribution with zero mean and unit standard deviation (Equation 1):

(Equation 1) $$x*=\frac{x-\mu }{\sigma }$$

where μ and σ are the mean and standard deviation of that feature computed on the training set, respectively. Standardization parameters are computed solely from the training set and then applied to the validation and test sets to prevent data leakage. The standardized dataset is used consistently across all models to guarantee fair comparison and compatibility with gradient-based optimization algorithms.

#### Machine learning models

##### Stacking ensemble method

Stacking is a meta-learning strategy that combines multiple base models with a meta-model to improve predictive performance.^42^ Unlike bagging or boosting, stacking allows the meta-model to learn from the outputs of the base models, thereby capturing complementary information across different models and reducing generalization error.^43^

This study selects five models that perform well on structured regression tasks as base models: XGBoost, CatBoost, LightGBM, deep neural networks (DNN), and random forest (RF).^44^ These models have distinct learning preferences. XGBoost and LightGBM adopt gradient boosting strategies and handle nonlinear relationships efficiently. CatBoost is robust to categorical features. DNN can approximate complex high-dimensional mappings. RF reduces variance by aggregating multiple decision trees. This diversity provides complementary decision boundaries for the stacking framework.

##### Meta-model: Linear regression and its variants—Ridge regression and elastic net

The meta-model in a stacking framework integrates the outputs of base models to enhance generalization and prevent overfitting. To balance interpretability and regularization capability, this study selects linear regression and two of its regularized variants—ridge regression and elastic net—as candidate meta-models.

Linear regression fits the relationship between meta-features and true values by minimizing the mean squared error (MSE) (Equation 2):

(Equation 2) $$MSE=\frac{1}{n}\sum_{i=1}^{n}{\left(y_{i}-\beta_{0}y-\sum_{j=1}^{p}\beta_{j}{\hat{y}}_{ij}\right)}^{2}$$

where *y*_*i*_ is the true coefficient of compressibility, ${\hat{y}}_{ij}$ is the prediction of the j-th base model for sample i, *β*_*j*_ are the model coefficients, n is the number of samples, and p is the number of base models.

Ridge regression introduces an L2 penalty term into the loss function to mitigate multicollinearity (Equation 3)^45^

(Equation 3) $${Loss}_{Ridge}=MSE+\lambda \sum_{j=1}^{p}\beta_{j}^{2}$$

where λ is the regularization strength, optimized via cross-validation.

Elastic net combines L1 and L2 penalties, enabling both coefficient shrinkage and feature selection (Equation 4).^46^

(Equation 4) $${Loss}_{ElasticNet}=MSE+\lambda_{1}\sum_{j=1}^{p}|\beta_{j}|+\lambda_{2}\sum_{j=1}^{p}\beta_{j}^{2}$$

In this study, grid search is employed to optimize λ_1_ and λ_2_, with RMSE in cross-validation as the objective to determine the optimal parameters. The most suitable meta-model is then selected for the stacking ensemble.

##### Hyperparameter optimization: Model tuning based on BOHB

ToHyperparameter configurations critically affect the performance of machine learning models. Conventional grid search and random search are inefficient in high-dimensional spaces and prone to local optima. To address these issues, the BOHB hybrid optimization algorithm is introduced.^47^ BOHB (Bayesian Optimization with HyperBand) combines the global search capability of Bayesian optimization with the adaptive resource allocation strategy of HyperBand. Unlike conventional Bayesian optimization, which evaluates all configurations with a full budget, BOHB terminates poorly performing configurations early, thereby reducing computational cost.^48^

The core iterative process of BOHB proceeds as follows. In each round, the algorithm randomly samples a new configuration with probability prand; otherwise, it builds a tree-structured Parzen estimator (TPE) based on historically observed data and selects the new configuration that maximizes the likelihood ratio (Equation 5):

(Equation 5) $$x_{t}∼\underset{x\in X}{\mathrm{argmax}}\frac{l\left(x\right)}{g\left(x\right)}$$

where l(x) and g(x) are the probability density functions of historically good and bad configurations, respectively. Subsequently, the HyperBand mechanism decomposes the training process of candidate configurations into multiple resource levels (e.g., numbers of iterations), dynamically eliminates poorly performing configurations, and concentrates computational resources on promising candidates until convergence.

##### Workflow of the ensemble model

AlthoughThe stacking process consists of the following three main steps.

Step 1: Train base models.

In each fold of 5-fold cross-validation, the base models optimized by BOHB are trained on 4-folds of the training set and generate predictions for the held-out fold. As each sample receives its prediction only from models not trained on that sample, the resulting meta-features are out-of-fold estimates that prevent information leakage from the base-model stage to the meta-model. Each base model produces predictions z_1_, z_2_, and z_3_ on the training set.

Step 2: Construct meta-features.

After 5-folds, the out-of-fold predictions of all base models are concatenated to form the *meta*-dataset Dmeta (Equation 6):

(Equation 6) $$D_{meta}={\left\{\left(z_{i1},z_{i2},z_{i3}\right),y_{i}\right\}}_{i=1}^{n}$$

where y_i_ is the true coefficient of compressibility of the i-th sample in the training set. The cross-validation mechanism ensures that the generation of meta-features does not rely on “future information”, effectively preventing information leakage and overfitting.

Step 3: Train the meta-model.

The selected meta-model is fitted to the relationship between meta-features and true values. Its loss function includes regularization terms (Equation 7):

(Equation 7) $$L\left(\beta \right)=\frac{1}{2n}\sum_{i=1}^{n}{\left(y_{i}-\sum_{j=1}^{p}z_{ij}\beta_{j}\right)}^{2}+\lambda_{1}\sum_{j=1}^{p}|\beta_{j}|+\frac{\lambda }{2}\sum_{j=1}^{p}\beta_{j}^{2}$$

where y is the true coefficient of compressibility, z are the predictions of the base models, β are the coefficients of the meta-model, and λ_1_ and λ_2_ are regularization strength parameters. When λ_1_ = 0 and λ_2_ = 0, the model reduces to linear regression; when λ_1_ = 0 and λ_2_ > 0, it reduces to ridge regression; when both are non-zero, it becomes elastic net.

Through the above three steps, the stacking model generates meta-features using cross-validation, integrates the complementary strengths of the base models via a regularized meta-model, and finally outputs the predicted coefficient of compressibility.

#### Cross-validation strategy

Two complementary cross-validation strategies were employed. For model development and hyperparameter tuning, 5-fold cross-validation was used within the training set to generate meta-features for stacking and to evaluate base model performance. The dataset was randomly split into training (85%) and test (15%) sets. All models were trained and evaluated over 30 independent runs with different random seeds (1–30).

For generalization assessment, spatial cross-validation (GroupKFold) was implemented to evaluate model performance on unseen sites.^49^ The 18 original engineering investigation sites served as the grouping variable, with all samples from each site assigned entirely to a single fold. 5-fold spatial cross-validation was performed, and R^2^, RMSE, and MAE were reported for each fold and as the overall mean.

#### Uncertainty quantification

A perturbation-based sensitivity analysis was performed using Monte Carlo simulation to evaluate model prediction stability under measurement errors.^50^ On the test set randomly sampled at 30% of the total test data, Gaussian noise with a standard deviation of ±5% of each feature value was added to the input. For each sample, 200 perturbed predictions were generated, and the 5th and 95th percentiles were calculated to construct the 90% prediction confidence interval. The prediction width, defined as the difference between the upper and lower bounds, was used to quantify uncertainty, with the mean width and its distribution across samples reported.

#### Sample size sensitivity analysis

The full dataset was first split into a fixed test set (15%) and a base training set (85%). The base training set was then randomly subsampled at proportions of 20%, 40%, 60%, 80%, and 100%. At each proportion, ten repeated random samplings were performed, and the mean and standard deviation of R^2^, RMSE, and MAE on both the training and test sets were calculated.^51^

#### Model interpretability methods

Feature importance was quantified using two complementary methods. Permutation importance was calculated by randomly shuffling each feature’s values and measuring the resulting degradation in model performance (R^2^) over 100 random permutations. SHAP (SHapley Additive exPlanations) values were computed using the KernelSHAP algorithm with the three base learners and ElasticNet meta-learner treated as a single predictive function, thereby attributing the ensemble output directly to the nine original input features.^52^ Mean absolute SHAP values were used to rank global feature importance, and SHAP beeswarm plots were used to visualize the direction and distribution of feature effects.

Accumulated Local Effects (ALE) and Partial Dependence Plots (PDP) were employed to visualize the functional relationship between individual features and model predictions.^53^^,^^54^ ALE was preferred as the primary method to avoid extrapolation bias caused by feature correlations. Three-dimensional PDPs and contour plots were constructed for selected feature pairs to reveal interaction effects.

### Quantification and statistical analysis

All statistical analyses were performed using Python (version 3.9). Statistical significance was defined as *p* < 0.05. Model performance was evaluated using the coefficient of determination (R^2^), root-mean-square error (RMSE), and mean absolute error (MAE). Pairwise model comparisons (e.g., three-model stacking vs. four-model stacking, stacking vs. CatBoost) were conducted using the Wilcoxon signed-rank test, a non-parametric paired difference test.
